# A Critical LRRK at the Synapse? The Neurobiological Function and Pathophysiological Dysfunction of LRRK2

**DOI:** 10.3389/fnmol.2020.00153

**Published:** 2020-08-27

**Authors:** Naila Kuhlmann, Austen J. Milnerwood

**Affiliations:** ^1^Faculty of Medicine, University of British Columbia, Vancouver, BC, Canada; ^2^Department of Neurology & Neurosurgery, Montreal Neurological Institute, McGill University, Montreal, QC, Canada

**Keywords:** Parkinson’s disease, LRRK2, synapse, neurotransmission, genetic mouse models, neuronal cultures

## Abstract

Since the discovery of *LRRK2* mutations causal to Parkinson’s disease (PD) in the early 2000s, the LRRK2 protein has been implicated in a plethora of cellular processes in which pathogenesis could occur, yet its physiological function remains elusive. The development of genetic models of LRRK2 PD has helped identify the etiological and pathophysiological underpinnings of the disease, and may identify early points of intervention. An important role for LRRK2 in synaptic function has emerged in recent years, which links LRRK2 to other genetic forms of PD, most notably those caused by mutations in the synaptic protein α-synuclein. This point of convergence may provide useful clues as to what drives dysfunction in the basal ganglia circuitry and eventual death of substantia nigra (SN) neurons. Here, we discuss the evolution and current state of the literature placing LRRK2 at the synapse, through the lens of knock-out, overexpression, and knock-in animal models. We hope that a deeper understanding of LRRK2 neurobiology, at the synapse and beyond, will aid the eventual development of neuroprotective interventions for PD, and the advancement of useful treatments in the interim.

## Introduction; A LRRK in the PD Arena

Parkinson’s disease (PD) is the second most common neurodegenerative disease, affecting 1–2% of the population by 65 years of age, and increasing to 4–5% by 85 years of age (de Lau and Breteler, 2006). Over 200 years after its initial clinical description (Parkinson, 2002), PD is still characterized primarily by its cardinal motor symptoms and the loss of dopamine (DA) neurons in the *substantia nigra* (SN). However, increasing recognition of non-motor symptoms and additional cell loss, such as in the cortex (MacDonald and Halliday, 2002) and thalamus (Henderson et al., 2000; Halliday, 2009), has highlighted the involvement of other neurotransmitter systems in early and later disease processes. While symptoms are significantly alleviated by interventions such as dopamine replacement therapies and deep brain stimulation, none of the current treatment options slow disease progression (reviewed in Oertel, 2017). It is hoped that advancing our understanding of PD etiology, including SN cell loss and beyond, will enable the production of neuroprotective treatments for PD. This requires uncovering etiological factors at the cellular level, and genetic models of PD are essential to this process. Moreover, they provide the opportunity to examine pathophysiological processes at various disease stages, while complimenting models of late-stage disease.

Although PD was long considered the archetypical non-genetic disease, it is now understood to arise from a complex interplay between environmental and genetic factors, with current estimates suggesting heritability underlies ~30% of PD risk (Keller et al., 2012; Goldman et al., 2019). The recognition of familial PD cases began ~20 years ago, with the identification of mutations, duplications and triplications in the *SNCA* gene encoding the α-synuclein (α-syn) protein (Polymeropoulos et al., 1997; Singleton et al., 2003; Chartier-Harlin et al., 2004). This discovery was a turning point for PD research, particularly given the later detection of α-syn in Lewy bodies (LB), the pathological protein inclusions found *post-mortem* in brains from people with PD and several related diseases now termed synucleinopathies (Goedert et al., 2013). In 2004, two separate studies identified multiple pathogenic mutations responsible for late-onset, autosomal-dominant parkinsonism—that they were all within the same gene, in the *PARK8* locus, really shook things up (Paisan-Ruiz et al., 2004; Zimprich et al., 2004). The sequence suggested the protein was a member of a newly described leucine-rich repeat kinase family (Manning et al., 2002); that protein, about which nothing was known, is LRRK2.

Perhaps the most thought-provoking aspect of LRRK2 PD was the discovery that clinical presentation, indistinguishable from “idiopathic” PD, is not always accompanied by traditionally expected pathology. While nigral cell loss is consistently observed in LRRK2 PD, α-syn-containing LBs are only found in about half of cases *post-mortem*; patients also present with ubiquitin-, tau-, or TAR DNA-binding protein 43-positive inclusions, or show nigral degeneration with no aggregate pathology (Zimprich et al., 2004; Rajput et al., 2006; Ujiie et al., 2012). This indicates that α-syn aggregation is not the cause of symptoms or nigral degeneration in half of LRRK2 PD, and therefore is not the cause of all forms of late-onset PD. That said, there is much evidence suggesting α-syn and LRRK2 proteins functionally interact, and that the dysfunction of either may disrupt a common physiological process, which eventually causes the disease to develop. Synucleins are one of the most abundant proteins in the brain and, as the name indicates, they are enriched at synapses (Maroteaux et al., 1988; Foffani and Obeso, 2018; Sulzer and Edwards, 2019). This enrichment is cell-type specific, with synuclein being highly expressed at excitatory terminals in the striatum by electron microscopy (Totterdell et al., 2004) and associated with structures positive for the vesicular glutamate transporter VGluT1 (Emmanouilidou and Vekrellis, 2016; Taguchi et al., 2016, 2019), but surprisingly not at TH expressing-nigral DA terminals (Emmanouilidou and Vekrellis, 2016; Taguchi et al., 2016). There is a clear consensus that α-syn is involved in regulating synaptic vesicle (SV) release, yet even after 30 years of progress, the underlying molecular physiology remains a matter of some debate (Sulzer and Edwards, 2019). Here we will review evidence accrued over the last 15 years that argues LRRK2, like synuclein, is also a regulator of synaptic physiology.

Since 2004, a host of animal models have examined loss-of-function, gain-of-function, and mutation-specific effects of LRRK2, implicating it in multiple cellular processes including neurite regeneration (Ramonet et al., 2011; Winner et al., 2011), autophagy (Albanese et al., 2019), endo-lysosomal sorting (MacLeod et al., 2013; Gómez-Suaga et al., 2014; Rivero-Ríos et al., 2019, 2020) and cytoskeletal dynamics (Parisiadou et al., 2009; Pellegrini et al., 2017). Although the literature on LRRK2’s role in PD etiology remains complex and inconclusive, membrane traffic is a common theme, and recent findings have also converged on the synapse as a key site of early pathophysiological change. Given that their intricate morphology and unique physiology require neurons to be uniquely dependent on endocytic and secretory processes, often at high frequencies and in the absence of cell replacement/regeneration, it is perhaps unsurprising if alterations to membrane traffic have negative effects in neurons, which may be tolerated in other cell types.

Several groups have linked LRRK2 to endocytic machinery (Shin et al., 2008; Matta et al., 2012; Arranz et al., 2015; Belluzzi et al., 2016), synaptic vesicle trafficking (Piccoli et al., 2011; Pan et al., 2017; Nguyen and Krainc, 2018), and altered synaptic transmission in multiple neuronal types (Tong et al., 2009; Beccano-Kelly et al., 2014, 2015; Sweet et al., 2015; Matikainen-Ankney et al., 2016; Volta et al., 2017). With emerging reports that LRRK2 functionally interacts with α-syn and other PD-linked proteins in axons and at the synapse (Lin et al., 2009; Inoshita et al., 2017; Mir et al., 2018; Novello et al., 2018; MacIsaac et al., 2020), therapeutic advancements may well depend on understanding how mutations in LRRK2 disrupt synaptic activity within the complex neural circuitry underlying PD. Many insights have come from invertebrate model systems, especially those overexpressing mammalian LRRK2; however, protostomes such as *C. elegans* and *Drosophila* have a single *LRRK* gene, of an ancient origin, which is more homologous to LRRK1 than LRRK2 (Marín, 2008). Thus, in the interest of brevity, the focus here is mostly restricted to mammalian LRRK2, as we discuss the evolution and current state of the literature placing LRRK2 at the synapse through the lens of knock-out, overexpression, and knock-in mammalian models.

## Where (and When) Does It LRRK?

In the initial studies investigating LRRK2 expression, mRNA was found in all human tissues examined, including heart, brain, placenta, lung, liver, skeletal muscle, kidney, and pancreas (Paisan-Ruiz et al., 2004; Zimprich et al., 2004). By RT-PCR, levels of LRRK2 mRNA were ~5-fold higher in the lung than the next highest tissue, the putamen of the striatum, which was ~2-fold that of other brain regions (Zimprich et al., 2004). While LRRK2 mRNA is highly expressed throughout embryonic development in the lung, kidney, spleen and heart (Zimprich et al., 2004; Biskup et al., 2006; Larsen and Madsen, 2009; Maekawa et al., 2010; Giesert et al., 2013), its expression within the CNS, primarily within the putamen of the striatum, increases drastically after birth. Northern blot showed a similar tissue pattern, with enrichment of LRRK2 transcripts in neocortex and putamen (Paisan-Ruiz et al., 2004). *In situ* hybridization studies in adult mouse brain confirmed LRRK2 mRNA was highest in the striatum and cortex (Melrose et al., 2006; Simón-Sánchez et al., 2006) but results varied for other regions including the hippocampus and SNpc. Galter et al. (2006) compared LRRK2 mRNA across the mouse, rat, and human *post-mortem* brain tissue, reporting high expression in striatal spiny projection neurons (SPNs), but no visible signal in SNpc dopamine neuron cell bodies. Thus, in human and mouse studies, LRRK2 mRNA is found in much of the circuitry implicated in PD but is not enriched (nor perhaps even present) in SNpc cell bodies; however, as highlighted below, mRNA transcript levels often do not correlate with protein abundance (reviewed in Liu et al., 2016).

The emergence of polyclonal LRRK2 antibodies (West et al., 2005), at the time not validated against LRRK2 knock-outs, provided the first glimpses of LRRK2 protein localization in the adult rat, mouse, and human brain. Widespread protein expression was found, with enrichment in the cortex and dorsal striatum, and low levels in the dopaminergic olfactory bulb and SNpc (Biskup et al., 2006). A direct comparison between LRRK2 mRNA and protein confirmed this; mRNA was high in regions receiving dopamine projections but absent in dopamine cell bodies, whereas LRRK2 protein was localized throughout the nigrostriatal pathway, including at low levels in the SNpc (Higashi et al., 2007). Elsewhere, no LRRK2 immunoreactivity was found in the olfactory tubercle, despite high mRNA expression, and the opposite pattern was seen in the thalamus (Melrose et al., 2006). A cross-comparison of the many available LRRK2 antibodies, tested against LRRK2 knock-outs, indicated extreme variability in the suitability of most across applications but did confirm LRRK2 protein expression in neuronal cell bodies (but not obviously in other cell types) of rodent cortex, striatum, and cerebellum (Davies et al., 2013), results that have since been replicated (West et al., 2014; Beccano-Kelly et al., 2015). Overall, reports from human, rodent and primate studies largely agree on striatal and cortical enrichment, with mixed findings in the SNpc (Biskup et al., 2006; Galter et al., 2006; Melrose et al., 2006; Simón-Sánchez et al., 2006; Higashi et al., 2007; Lee et al., 2010; Mandemakers et al., 2012; Davies et al., 2013). LRRK2 protein is also clearly found in other tissues; while not directly quantified between tissues, similar LRRK2 protein levels have been found in mouse lung, kidney, spleen, and brain (Mir et al., 2018), in agreement with the original mRNA observations (Zimprich et al., 2004). In terms of cell type, beyond neurons, abundant LRRK2 protein is found in mouse embryonic fibroblasts (Mir et al., 2018), and human neutrophils and monocytes (Fan et al., 2018; Mir et al., 2018; Atashrazm et al., 2019). Interestingly, a recent study demonstrated low levels of LRRK2 protein in human induced pluripotent stem cell (IPSC)-derived macrophages and microglia that were highly increased upon activation by IFN-γ, suggesting CNS stress and inflammation will upregulate LRRK2 in brain glial cells (Lee et al., 2020).

The elucidation of LRRK2’s developmental profile in the brain was another important advance; analyses of LRRK1/LRRK2 mRNA and protein expression found LRRK2 was expressed primarily in neurons at birth and increased in the first postnatal week particularly within cortex, striatum and olfactory bulb (Giesert et al., 2013). Interestingly, these first postnatal weeks are also marked by synaptogenesis, especially in the striatum and cortex (Mensah, 1982; Ishikawa et al., 2003). These results have been confirmed, with LRRK2 protein being present by embryonic day 15 in the cortex (Higashi et al., 2007; Beccano-Kelly et al., 2014), and levels rising >5 fold over 3 weeks in the postnatal brain and up to 21 days *in vitro* (DIV21) in cortical/hippocampal neuron cultures (Piccoli et al., 2011; Beccano-Kelly et al., 2014).

Expression analyses were paralleled by attempts to identify LRRK2’s subcellular localization. Fractionation studies found that LRRK2 was enriched in microsomal, synaptic vesicle-enriched, and synaptosomal cytosolic fractions in rat brain tissue (Biskup et al., 2006). LRRK2 also separated with markers of synaptic plasma membrane vesicles in mouse brain extracts (Hatano et al., 2007). Immunocytochemical fluorescence studies in neuronal cultures suggested LRRK2 localized to lysosomes, mitochondria, and microtubules (Biskup et al., 2006), in addition to Golgi and the synaptic vesicle (SV) marker synaptotagmin-1 (Hatano et al., 2007). Unfortunately, LRRK2 antibodies have almost universally failed tests of specificity against knock-out samples in immunofluorescence experiments (Davies et al., 2013). To avoid the confounds of LRRK2 antibody specificity, Schreij et al. (2015) gene-edited an HA-tagged LRRK2, and found it colocalized with clathrin-light chains and the early endosomal marker EEA1. As this was done in COS-7 cells, this possibly included synaptic endosomes, and other studies have placed LRRK2 at synaptic endosomes, in association with Rab5 (Shin et al., 2008; Yun et al., 2015; Inoshita et al., 2017). Such literature provided support that LRRK2 has a seat at the synapse, but what of functional observations?

At this juncture, it is important to note some parameters of synaptic maturation (expertly reviewed in Sala and Segal, 2014; Kavalali, 2015; Andreae and Burrone, 2018; Chanaday and Kavalali, 2018), given that one of the most important, but underappreciated, confounds to the interpretation of many LRRK2 studies is the maturation state of the chosen system. Excitatory synapses develop their specialized synaptic structures as they mature, over a similar timeframe both *in vivo* and in rodent primary cultures of cortex and hippocampus, in which many pertinent observations have been reported. In cultures, immature postsynaptic protrusions, filopodia, and thin spines re-appear on dendrites between 4 and 7 days *in vitro* (DIV4–7) after excitatory neurites have regenerated, and new contacts begin to form between axons and dendrites (Papa et al., 1995; Boyer et al., 1998; Takahashi et al., 2003). As the postsynaptic structures mature, they become shorter, fatter, and mushroom-like. By DIV14 the number of postsynaptic protrusions doubles, being ~50:50 immature- and mature-looking; the number again doubles by DIV21, at which point densities stabilize, and 80–90% of protrusions exhibit a mature morphology (Papa et al., 1995; Boyer et al., 1998; Takahashi et al., 2003). Unlike hippocampal and cortical cultures composed of predominantly excitatory cells, GABAergic medium-sized spiny striatal projection neurons (>90% of striatal cells) do not develop a complex dendritic architecture or dendritic spines if grown in monocultures (Segal et al., 2003; Kaufman et al., 2012; Burguière et al., 2013). However, if co-cultured with glutamatergic neurons, they form a great many excitatory synapses over the same time-frame, and to approximately the same extent as *in vivo* (Segal et al., 2003; Tian et al., 2010; Randall et al., 2011; Kaufman et al., 2012; Milnerwood et al., 2012; Burguière et al., 2013; Lalchandani et al., 2013; Penrod et al., 2015). These patterns of synaptic maturation up to ~DIV21 are matched by immunostaining of synaptic marker cluster density, colocalization of pre- and postsynaptic markers, biochemical quantification of synaptic proteins, and synaptic activity (Levinson and El-Husseini, 2005; Arstikaitis et al., 2008, 2011; Han and Stevens, 2009; Beccano-Kelly et al., 2014; Harrill et al., 2015). Furthermore, as mentioned, this is the same temporal expression pattern as that for LRRK2 protein (Piccoli et al., 2011; Beccano-Kelly et al., 2014).

Such maturation is dependent on appropriate patterns of presynaptic release, which also requires time to mature (reviewed in Kavalali, 2015); young, developing synapses (<DIV8) lack a readily releasable vesicle pool (RRP), but can spontaneously release glutamate through a slow recycling pool of vesicles (Mozhayeva et al., 2002). The frequency of spontaneous currents in these developing synapses can be increased by strong depolarization (e.g., high extracellular [K^+^]), but not by action potentials or hypertonic sucrose (Mozhayeva et al., 2002). In contrast, older synapses (>DIV12) respond to strong depolarization, action potentials, and hypertonic sucrose (Mozhayeva et al., 2002; Andreae et al., 2012). Thus, as synapses mature beyond the first 2 weeks *in vitro*, they utilize different means of release, dependent on different forms of vesicle cycling, and begin to become structurally and functionally mature, a process that appears to plateau at ~DIV21 (reviewed in Kavalali, 2015).

## No LRRK2, No Problem? Silencing, Redundancy, and Target Validation

Early LRRK2 knock-out (LKO) models sought to examine whether loss-of-function recapitulated parkinsonian motor dysfunction, DA loss, and α-syn accumulation. Despite reports of peripheral phenotypes, most notably within the kidney and lung (Tong et al., 2010; Herzig et al., 2011), LKO mice, and even wild-type (WT) mice subject to acute LRRK2 knock-down (Volta et al., 2015a), do not present with overt PD-like phenotypes, and generally seem normal in terms of behavior and neurophysiology (Andres-Mateos et al., 2009; Lin et al., 2009; Hinkle et al., 2012; Beccano-Kelly et al., 2014, 2015; Volta et al., 2015a). This suggests loss-of-function is unlikely to explain PD pathology or etiology, and that LRRK2 may itself be a safe and attractive therapeutic target if ablated specifically within the CNS to avoid peripheral tissue damage. So, if LRRK2 can be eliminated without dire consequence, what clues do we have from deletion studies as to the neurophysiology of LRRK2?

Early reports comparing neurite length in very young (<7 DIV) cultures (Parisiadou et al., 2009; Dächsel et al., 2010), or soon after knock-down in older cultures (MacLeod et al., 2006; Meixner et al., 2011), suggested that LKO neurites show elevated growth. However, a more recent longitudinal study over 3 weeks *in vitro* found no difference in axon outgrowth (DIV3) or total dendritic length (up to DIV21) in LKO neurons (Sepulveda et al., 2013). That said, when examining neurites by time-lapse imaging, Sepulveda et al. (2013) found increased axonal and dendritic motility in LKO neurons, depending on the growth substrate. This may be indicative of less mature/stable processes in LKO, and explain the differences observed at single time points in very young neurons (Parisiadou et al., 2009; Dächsel et al., 2010). Together, these reports could be interpreted as evidence for slightly slower maturation in LKO scenarios, over the first couple of weeks *in vitro*. Nevertheless, a recent study reported forebrain atrophy and reduced dendritic complexity in SPNs of 12- (but not 2-) month-old LKO mice, accompanied by changes in nuclear morphology and some motor impairment compared to WT mice, in contrast to hyperactivity observed in younger LKO mice (Chen et al., 2020). Thus, investigating age-dependent changes in dendritic morphology in an *ex vivo* context may warrant further attention.

Functional investigation of individual synapses has been conducted by vesicle dye-imaging experiments to examine exocytosis and endocytosis in the absence of LRRK2. On the first approximation, these have also yielded conflicting results. In the seminal study, siRNA-mediated LRRK2 knock-down in cultured rat hippocampal neurons did not affect synaptic exocytosis at DIV14, but did slow/impair endocytosis; however, this also occurred with overexpression of WT and mutant (G2019S or R1441C) LRRK2 (Shin et al., 2008). Slowed/reduced SV endocytosis (and normal exocytosis) was also observed at the neuromuscular junction in a *Drosophila* Lrrk KO (Matta et al., 2012), and in striatal neurons from LKO rats at ~DIV11 (Arranz et al., 2015). The latter was conducted in striatal mono-cultures, which usually yield 70–90% GABAergic neurons (Shehadeh et al., 2006; Kaufman et al., 2012); thus, synaptic vesicle endocytosis was likely impaired at developing GABAergic terminals (Arranz et al., 2015). In contrast to mono-cultures, when grown in co-culture with glutamatergic neurons, striatal neurons develop a more complex dendritic architecture, acquire their eponymous dendritic spines, and benefit from increased pro-survival signaling through glutamate receptors (Segal et al., 2003; Kaufman et al., 2012; Milnerwood et al., 2012). This may explain why a study similar to Arranz et al. (2015), but using older DIV14–17 cells in cortico-striatal co-cultures from LKO mice, found unaltered exocytosis but modestly increased endocytosis at striatal GABAergic synapses (Maas et al., 2017). Thus, the cellular environment may dictate LKO phenotypes, especially the age/maturity of the neuronal culture, and the amount of LRRK2 that should be expressed at any given age. Moreover, the response to LRRK2 knock-out may differ between cell types e.g., in the same study that found increased endocytosis in older co-cultured LKO striatal neurons, no changes to endocytosis (or exocytosis) were seen in hippocampal cultures (Maas et al., 2017).

Regardless of how LKO might disturb the vesicle cycle, one would expect a consequence for synaptic transmission; however, on the surface, investigations of synapse function in LKO have also appeared somewhat contradictory. In the same study that described reduced endocytosis in cultured LKO mouse striatal neurons, Arranz et al. (2015) found no effect on spontaneous excitatory postsynaptic currents (sEPSCs; action potential-dependent currents included) in hippocampal neurons (aged DIV7–12). This lack of alteration is consistent with intact endocytosis in other similarly aged LKO hippocampal preparations (Maas et al., 2017). More mature (DIV21) LKO cortical cultures, which are similar to hippocampal cultures, also had no alterations to synapse markers or miniature EPSCs (mEPSCs; quantal glutamate release only, with action potentials blocked; Beccano-Kelly et al., 2014). Hypertonic sucrose can be used to stimulate release from the readily releasable pool, driving an increase in sEPSC frequency; Arranz et al. (2015) found that this effect was absent in DIV7–12 LKO hippocampal cells. This could reflect impaired release, but might also result from LKO synapses maturing slightly slower, as this form of release is normally absent in cultures at <DIV8 (Mozhayeva et al., 2002).

Together, experiments in glutamatergic cell cultures suggest no gross alterations to neurite growth, endocytosis, or basal synaptic activity due to LRRK2 germ line knock-out, especially in more mature systems. However, differences in LKO throughout early maturation may indicate a developmental delay resulting from the absence of LRRK2. This is supported by functional experiments in brain slices from LKO mice, where decreased glutamate transmission has been observed in striata of postnatal day (P) 15 mice (Parisiadou et al., 2014), but not in slices from >3-month-old animals (Beccano-Kelly et al., 2015). Similarly, no differences were found in glutamate transmission in hippocampal slices from 3-week-old LKO mice (Maas et al., 2017), nor in dopamine release in 18-month-old animals (Hinkle et al., 2012). Overall, while loss-of-function studies first implicated LRRK2 in synaptic transmission, the weight of evidence suggests that deleting LRRK2 results in modest and transient effects, far from those observed in PD.

## More LRRK2, More Problems?

Given a lack of strong behavioral or degenerative phenotypes when deleting LRRK2, a logical conclusion is that pathophysiological mutant effects result from gain-of-function. The past decade of research on over-expression (OE) models supports this, but with a twist in the story; wild-type LRRK2 overexpression imparts PD-relevant changes in behavior, dopaminergic neurons, and even α-synuclein accumulation in mice, but does not produce nigral cell loss. This is similar to other PD genetic models based on α-synuclein, where the vast majority of models, including wild-type α-syn OE mice, also lack cell death (Giasson et al., 2002; excellently reviewed in Chesselet and Richter, 2011).

The first report of an organismal gain-of-function was in *Drosophila*, where expressing full-length human LRRK2 produced a 28% loss of dopamine neurons, a progressive decline in climbing ability, and premature mortality (Liu et al., 2008). This has been replicated in other *Drosophila* studies (Islam et al., 2016), but results from mouse LRRK2 OE models are more mixed. One study reported no pathology or motor phenotype in 12-month-old mice overexpressing human wild-type (hWT) LRRK2 at 8–16-fold endogenous levels (Lin et al., 2009). However, when combined with the A53T α-syn mutation, hWT-LRRK2 OE promoted the accumulation of α-syn in 1-month-old mice, impaired microtubule dynamics, and caused Golgi fragmentation, suggesting an interaction whereby overabundant LRRK2 accelerates α-syn-mediated neurodegeneration (Lin et al., 2009).

Studies using bacterial artificial chromosomes (BACs) to overexpress hWT-LRRK2 in mice resulted in a reduction in striatal DA tone, measured by microdialysis (Melrose et al., 2010; Beccano-Kelly et al., 2015), accompanied by either no behavioral deficit (Li et al., 2009; Melrose et al., 2010) or (in larger cohorts) hypoactivity and impaired recognition memory at 6 (Beccano-Kelly et al., 2015) and 12 months (Volta et al., 2015a). In contrast, BAC-mediated overexpression of murine WT LRRK2 led to progressive hyperactivity and improved motor performance, likely related to a ~25% increase in evoked extracellular DA (Li et al., 2010). BAC models include human or murine regulatory elements, consequently driving variable expression levels and/or patterns, which may underlie differences in behavior and regulation of dopamine homeostasis. Thus, overexpressing LRRK2 in a human-specific pattern produced some parkinsonian-like phenotypes in these rodents, whereas overexpressing LRRK2 in a mouse-specific pattern led to hyperdopaminergia and hyperactivity. These studies again show that the consequences of LRRK2 manipulations depend upon the cell type being studied. In light of that, selectively overexpressing hWT-LRRK2 in dopamine neurons resulted in moderate hyperactivity, as well as elevated dopamine release in young mice (Liu et al., 2015).

Dopamine alterations from LRRK2 overexpression are accompanied by dysfunction at glutamate synapses; while there were no basal electrophysiological differences observed in evoked glutamate release onto striatal neurons, concomitant dopamine release negatively tuned glutamate release onto SPNs of hWT-LRRK2 OE mice, an effect eliminated by presynaptic D2 receptor (D2R) blockade (Beccano-Kelly et al., 2015). This may have been in part due to elevated presynaptic D2R protein, but similar changes were not observed at nigral terminals, suggesting the increase could be specific to glutamate synapses (Beccano-Kelly et al., 2015). While neuromodulation appears altered, a direct effect of LRRK2 overexpression on the glutamatergic release is less clear: there were similarly no basal differences in synaptic transmission in hippocampal slices from BAC hWT-LRRK2 OE mice compared to non-transgenic animals (Sweet et al., 2015), but cortical cultures show a small increase in synapse density and a non-significant trend to increased spontaneous release (Beccano-Kelly et al., 2014). Overall, an overabundance of normal LRRK2 confers more pathophysiological changes than eliminating it, but effects on behavior, dopamine release, and glutamate transmission are dependent on the expression pattern (e.g., mouse vs. human BAC) and context (e.g., adult brain slice vs. culture).

Many of the aforementioned studies also examined overexpression of LRRK2 harboring pathogenic mutations, particularly those within the kinase or Roc-GTPase domains. In *Drosophila*, overexpressing human LRRK2 with either G2019S (Liu et al., 2008) or R1441C (Islam et al., 2016) mutations led to a more severe loss of TH-positive neurons and climbing ability, and caused earlier mortality than hWT-LRRK2 OE. Overexpressing the R1441C mutation additionally downregulated SNARE proteins SNAP-25 and syntaxin 1A, as well as exocytosis-related proteins synaptotagmin-1 and Rab3, suggesting that the resulting pathology may be linked to synaptic vesicle dynamics (Islam et al., 2016). In mice, overexpressing human LRRK2 with the G2019S mutation (hG2019S-LRRK2) did not worsen the A53T α-syn-mediated neurodegeneration already present when overexpressing hWT-LRRK2, suggesting that the pathological interaction is not entirely dependent on kinase activity (Lin et al., 2009). However, a subtle motor phenotype emerged, where hG2019S-LRRK2 OE mice exhibited increased ambulatory activity at 12 months when compared to hWT-LRRK2 OE and non-transgenic mice. Similarly, increased exploratory behavior was observed in BAC hG2019S-LRRK2 OE mice, in contrast to a lack of phenotype in hWT-LRRK2 OE mice (Melrose et al., 2010). In line with this, Tet-inducible G2019S-LRRK2 OE in rats led to enhanced locomotor activity at 12 months, attributed to impaired dopamine reuptake (Zhou et al., 2011). Elsewhere, two studies (Li et al., 2010; Liu et al., 2015) found no behavioral abnormalities with G2019S-LRRK2 OE, despite significant alterations to DA axon terminals and reductions in evoked striatal DA, compared to the increased motor activity and DA release observed with hWT-LRRK2 OE.

Part of the confusion between these studies may be explained by context and age-dependent phenotype presentation. Young mice overexpressing hG2019S-LRRK2 OE showed increased exploration and normal cognitive performance at 6 months, an age at which hWT-LRRK2 OE mice were impaired in both tasks, but mutant OE went on to display similar cognitive deficits at 12 months (Volta et al., 2015a). Chen et al. (2012) provide further evidence of hypoactivity in 12- to 16-month-old G2019S-LRRK2 OE mice, which was rescued by L-DOPA treatment. Species and overexpression levels also factor in: a recent study in rats found that while overexpressing the C-terminal domain of G2019S-LRRK2 in the SNpc *via* lentivirus did not affect the number of DA neurons, the higher expression level obtained by adeno-associated virus (AAV) led to 30% TH neuron loss within 6 months (Cresto et al., 2020). Importantly, this did not lead to a concomitant motor deficit within the examined time frame, highlighting a disconnect between cell loss and motor phenotypes (Cresto et al., 2020).

Regardless of how motor and behavioral phenotypes present, these studies generally converge on altered dopamine transmission, likely due to altered synaptic vesicle endo- and exocytosis. In support of this, selective hG2019S-LRRK2 overexpression in DA neurons resulted in behavioral deficits, increased pathologic phosphorylation of α-syn, reduced synaptic vesicle number, and increased clathrin-coated vesicles (CCVs) at DA terminals (Xiong et al., 2018). A closer look at endo- and exocytic machinery in OE models further implicates a role for LRRK2. Recent work from Pan et al. (2017) suggest that the G2019S mutation disrupts the synaptic vesicle cycle in cultured neurons and converges with another PD-linked protein, synaptojanin-1 (synj1), a key mediator of clathrin coat removal from endocytosed synaptic vesicles. Notably, G2019S-LRRK2 impaired endocytosis specifically in midbrain neurons, and enhanced exocytosis in hippocampal and cortical neurons (Pan et al., 2017). While this suggests neuron-specific effects and provides a potential mechanism for the selective vulnerability of DA neurons (Pan et al., 2017), the assays were conducted in immature (~DIV7) cultures in which there are normally low levels of endogenous LRRK2. In contrast, another group found that the G2019S mutation increased LRRK2-dependent phosphorylation of Snapin, a presynaptic SNARE protein involved in exocytosis, thereby decreasing the readily releasable pool and exocytotic release in slightly older (DIV18) hippocampal neurons (Yun et al., 2013).

Further evidence of non-dopaminergic alterations induced by G2019S-LRRK2 overexpression comes from reports of increased synaptic transmission and altered synaptic plasticity in acute hippocampal slices (Sweet et al., 2015), and upregulation of the 5-HT1A serotonin receptor, resulting in anxiety and depression-like behavior (Lim et al., 2018). Overall, overexpression of the G2019S mutation results in more dramatic neural dysfunction than overexpressing higher levels of WT LRRK2, including altered endo- and exocytosis (Pan et al., 2017), dopamine axon terminal damage (Chen et al., 2012; Liu et al., 2015), cytoskeletal changes (Parisiadou et al., 2009; Winner et al., 2011), synaptic plasticity deficits (Sweet et al., 2015), phosphorylated tau accumulation (Melrose et al., 2010; Chen et al., 2012) and mitochondrial dysfunction (Ramonet et al., 2011; Liu et al., 2015).

As the most common mutation, G2019S has been more extensively modeled, but LRRK2 mutations in the Roc-GTPase domain cause similar pathophysiological changes. R1441G-LRRK2 OE in mice results in progressive motor deficits that are responsive to L-DOPA, and fragmentation of dopaminergic axon terminals (Li et al., 2009). The initial report suggests a phenotype that is arguably more severe than those resulting from G2019S-LRRK2 overexpression, but subsequent studies reported much milder motor effects (Bichler et al., 2013; Dranka et al., 2013). Overexpressing WT, G2019S- and R1441C-LRRK2 has been directly compared, with mutation-specific effects being found in LRRK2 expression pattern, dopamine turnover, neuronal degeneration, and motor phenotype (Ramonet et al., 2011). Moreover, in mouse models, both mutations appear to converge on synaptic vesicles endocytosis. Nguyen and Krainc (2018) recently determined that patient iPSC-derived dopamine neurons from both R1441C/G and G2019S mutation carriers have increased phosphorylation of auxilin (a protein acting downstream from synj1), impaired endocytosis, and reduced SV density. Together, these studies suggest overexpression of pathogenic LRRK2 mutations produces similar behavioral and physiological effects, that are distinct from, or more pronounced than, the effects of WT overexpression.

Overexpression models have produced a wealth of information regarding the possible pathophysiological processes in LRRK2-PD, but they have been mired by confounding variables that makes interpretation, and especially the comparison between models, very difficult. In particular, expression levels have varied from ~2-fold (Melrose et al., 2010; Beccano-Kelly et al., 2015; Volta et al., 2015a) to ~16-fold (Lin et al., 2009) and effects are distinct between human and mouse regulation of expression, as well as between constitutive vs. acute overexpression (Zhou et al., 2011). Lastly, much-employed BAC transgenesis has the huge advantage of including the relevant regulatory elements for LRRK2 gene expression, but also engenders species- and cell type-dependent expression patterns, in addition to caveats of random gene insertion (with each construct), and the presence of endogenous LRRK2 expression (Daniel and Moore, 2014). Such confounds were the impetus for the development of germ-line “knock-in” models, in which disease mutations are expressed in the endogenous *Lrrk2* gene, enabling examination of mutation-specific effects with the point mutations being the only variable.

## Recapitulation of Genetic Predisposition by Knock-in of PD Mutations

The first LRRK2 mutant knock-in (KI) mice were produced by introducing the R1441C mutation into the endogenous LRRK2 Roc domain (Tong et al., 2009). These R1441C KI mice displayed grossly normal motor behavior and no nigral TH or cell loss. However, the hyperlocomotive response to amphetamine was absent in these animals, and locomotor inhibition by D2 agonism was reduced, suggesting an altered dopamine system (Tong et al., 2009). The authors concluded that not only was dopamine transmission impaired, but nigral neuron firing was also much less sensitive to inhibition by dopamine and dopamine agonists (Tong et al., 2009). Aging to >24 months revealed emergent phenotypes in R1441C KI mice, where subtle motor and prodromal PD-like alterations were detected, including impaired gait and olfaction; however, another study found no signs of nigral cell loss, changes in SPN morphology, or endo- or exocytosis in cultured hippocampal neurons (Giesert et al., 2017).

Additional experimental perturbation of the dopamine system is also required to uncover a motor phenotype in R1441G knock-in mice. Acute catecholamine depletion by reserpine led to greater locomotive impairment and failed recovery in R1441G KI compared to WT mice (Liu et al., 2014). In an *in vitro* assay of dopamine uptake, R1441G-LRRK2 synaptosomes trended toward less uptake at 10 months of age, and a significant reduction in mutant dopamine uptake was observed in reserpinated mice, suggesting perturbed DA homeostasis (Liu et al., 2014). This was thought to be due to impaired dopamine transporter (DAT) function, given that isolated synaptosomes from 3- and 18-month-old mutant mice showed reduced DA uptake following reserpine depletion (Liu et al., 2014). Together the data suggest that endogenous expression of LRRK2 Roc-GTPase mutations confers latent motor impairment and alterations to striatal dopamine regulation/homeostasis; however, it must be noted that other neurotransmitter systems have not yet been addressed in this context.

Herzig et al. (2011) presented the first G2019S-LRRK2 knock-in (GKI) mice, finding no pathological or locomotor differences at 5 months of age, even after cocaine administration. Elsewhere, the same animals were shown to exhibit a basal hyperactive phenotype, beginning at 6 months and persisting to up to 15 months of age, which was reversed by LRRK2 kinase inhibition (Longo et al., 2014). Other independent reports of GKI mice found a subtle and transient enhancement of motor activity (Yue et al., 2015) and exploratory rearing in a cylinder test (Volta et al., 2017). Although reported motor phenotypes appear to be subtle and context-dependent, several alterations to the dopaminergic system have been observed. *A* >50% reduction of striatal dopamine levels and stimulated release was seen by *in vivo* microdialysis in 12-month-old, but not 6-month-old, G2019S knock-in mice (Yue et al., 2015). This was thought to be due to impaired exocytosis, given that dopamine metabolites were not altered, and reverse DA transport was intact (Yue et al., 2015). However, when striatal dopamine release was directly assayed in brain slice by fast-scan cyclic voltammetry, no impairment was found in mice aged >12 months (Volta et al., 2017). Conversely, younger mice (3 months) exhibited increased dopamine release with repeated stimuli, and slower single response decay, indicating an elevated extracellular lifetime of dopamine (Volta et al., 2017). Slower responses were independent of DAT clearance, being maintained when blocking DAT (Volta et al., 2017); further, DAT levels and activity are not impaired but are higher in older G2019S knock-in mice (Longo et al., 2017; Volta et al., 2017). Thus, a persistent augmentation in DA release may be masked by increased DAT clearance in older GKI mice.

Evidence for the G2019S mutation perturbing other neurotransmitter systems has also emerged. Increased glutamate miniature event frequency was reported in 3-week-old cortical neurons cultured from G2019S KI mice, with no change in the density of synaptic markers, likely reflecting an increase in the probability of release (Beccano-Kelly et al., 2014). This was paralleled by increased spontaneous event frequency in striatal slices from the same G2019S KI mice, present at 3 months but reduced to WT levels by 12 months (Volta et al., 2017). A transient effect was also observed independently in slices from similar P21 GKI mice, with elevated glutamate event frequency primarily through cortical neuron firing; this was not seen in LRRK2 kinase-dead mice, was normalized by LRRK2 kinase inhibition, and was absent in slices from older animals (Matikainen-Ankney et al., 2016). Neither study found differences in glutamatergic synapse markers (Matikainen-Ankney et al., 2016; Volta et al., 2017), again suggesting that presynaptic release, not synapse number, was the source of elevated event frequency.

G2019S knock-in mice also exhibit postsynaptic alterations. A significant increase in SPN dendritic spine head width was observed in the dorsolateral striatum (Matikainen-Ankney et al., 2016), along with reduced calcium-permeable AMPA receptors in the ventral striatum/nucleus accumbens (Matikainen-Ankney et al., 2018). In addition to differences in basal transmission, striatal LTP was absent in G2019S knock-in mice aged <2 months (Matikainen-Ankney et al., 2018). Altered responses to dopamine agonists and antagonists have also been found at glutamatergic synapses on SPNs (Volta et al., 2017; Tozzi et al., 2018); D2 dopamine receptor agonism had an augmented effect on negatively tuning dopamine release in slices from young GKI mice, but glutamatergic synapses were relatively insensitive to D2 agonism and antagonism (Volta et al., 2017). In contrast, others found exaggerated responses to D2 agonism in older G2019S knock-in mice (Tozzi et al., 2018). Such changes to long- and short-term plasticity at excitatory synapses may underlie some cognitive and psychiatric phenotypes observed in LRRK2 mouse models (Volta et al., 2015a; Adeosun et al., 2017; Matikainen-Ankney et al., 2018; Guevara et al., 2020).

Together, motor phenotypes in LRRK2 mutant knock-in mice appear subtle, age- and context-dependent, but hyperactivity has been consistently reported. A lack of gross motor dysfunction is arguably appropriate for a model of PD etiology, early pathophysiology, and pre-motor dysfunction (see “Conclusions and Future Directions” section), especially so over the limited lifespan of a mouse. The weight of evidence suggests that LRRK2 mutations result in dysfunction at dopamine and glutamate synapses, and likely in other neurotransmitter systems, such as GABA (Beccano-Kelly et al., 2014) and serotonin (Lim et al., 2018). Interestingly, synaptosomes prepared from the striatum or cerebral cortex of the same animal revealed opposite effects of the G2019S mutation, as well as LRRK2 kinase inhibition, on dopamine and glutamate release (Mercatelli et al., 2019), providing further evidence that LRRK2’s actions are brain region-, synapse-, and age-specific. Overall, the literature provides a strong argument for further study of pathophysiological changes at the circuit level, including in dopamine, glutamate, and GABA transmission. This may be particularly pertinent in the striatum, given that is where these systems functionally interact and is precisely “where the action is” in PD pathogenesis.

## Molecular Interactors and the LOCI of LRRK2 Dysfunction

More than a decade of research has provided a wealth of evidence for synaptic LRRK2 function, but the underlying mechanisms remain unclear. Many potential binding partners and substrates have been identified, although some may be a result of forced *in vitro* interactions that do not occur physiologically in neurons, let alone at synapses. As in all fields of modern neuroscience, progress on the molecular cell biology of LRRK2 has been hampered by poorly selective LRRK2 antibodies and kinase inhibitors, although the joint effort to standardize such resources by academia, industry, and non-profit organizations set an example for other research communities (Davies et al., 2013; Ito et al., 2016; Steger et al., 2016; Mir et al., 2018). In no small part thanks to this, many promising candidates have emerged.

**Figure 1:**
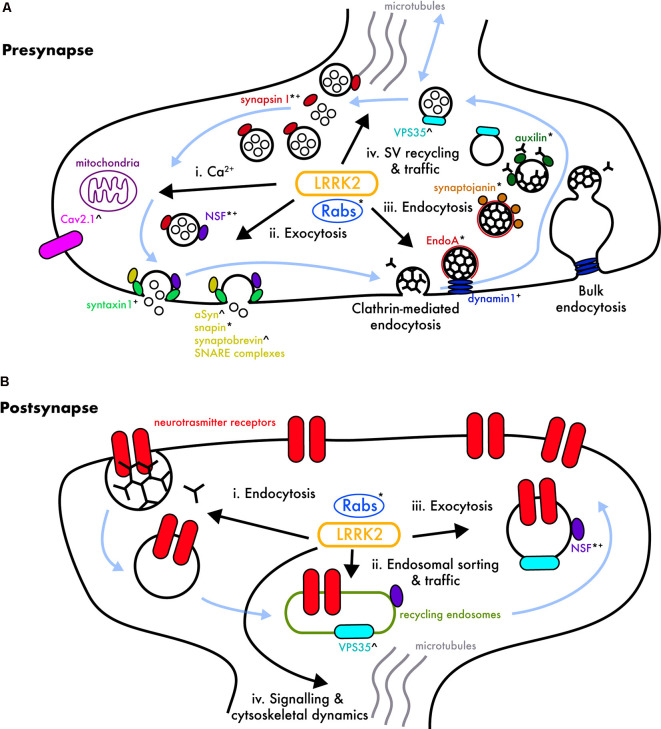
LRRK2’s potential involvement in pre- and post-synaptic pathways. **(A)** Cartoon of a generalized presynaptic terminal showing processes in which LRRK2 has been implicated. Synaptic vesicles (SVs; large black circles), and their cycling (blue arrows) regulate the loading of neurotransmitters (small black circles), which are released by Ca^2+^-dependent fusion at the synaptic active zone into the synaptic cleft. SVs are retrieved by clathrin-mediated endocytosis (black triskelia), and/or bulk endocytosis, and subsequent clathrin coating/uncoating. Vesicles are recycled through endosomal intermediates back into the vesicle cycle. Various members of the Rab GTPase family, many of which are LRRK2 substrates, regulate nearly all steps of this cycle (see text), and other major molecular regulators are named, and marked, depending on the proposed LRRK2 association; putative LRRK2 kinase substrates*, physical LRRK2 binding^+^, and functional interactions with a mechanism to be determined^∧^. **(i)** Calcium (Ca^2+^) flux and buffering may be altered by pathogenic LRRK2 mutations, as LRRK2 regulates Ca_V_2.1 voltage-gated calcium channels (Bedford et al., 2016), and mitochondrial homeostasis (Cherra et al., 2013; Verma et al., 2017); disruption of these processes would create downstream effects on Ca^2+^-dependent vesicular exocytosis. **(ii)** Exocytosis is dependent on numerous proteins that regulate synaptic vesicle availability, traffic, and active zone SNARE complex assembly/disassembly; N-ethylmaleimide sensitive factor (NSF; Piccoli et al., 2014; Belluzzi et al., 2016), syntaxin 1 (Piccoli et al., 2011, 2014; Islam et al., 2016), α-syn (Bieri et al., 2019; MacIsaac et al., 2020), and snapin (Yun et al., 2013) have been linked to LRRK2, in addition to Rab3 (not shown), which colocalizes with α-syn and maybe an LRRK2 substrate (reviewed in Shi et al., 2017). **(iii)** Classical clathrin-mediated- and bulk-endocytosis both appear important in mature synaptic terminals (Clayton and Cousin, 2009; Clayton et al., 2010; Gross and von Gersdorff, 2016; Chanaday and Kavalali, 2018). LRRK2 is implicated in synaptic endocytosis *via* a functional interaction with Endophilin A (Matta et al., 2012; Ambroso et al., 2014; Arranz et al., 2015; Soukup et al., 2016), synaptojanin (Piccoli et al., 2014; Islam et al., 2016; Pan et al., 2017), auxilin (Nguyen and Krainc, 2018), and dynamin1 (Piccoli et al., 2011; Stafa et al., 2013). Early endosome formation, mediated by potential LRRK2 substrate Rab5b (Shin et al., 2008; Yun et al., 2015), is required for the transport of clathrin-dependent endosomes (reviewed in Shi et al., 2017). **(iv)** SV trafficking and recycling may involve LRRK2 in concert with VPS35 (Inoshita et al., 2017; Mir et al., 2018), Rab29 (aka Rab7L1; MacLeod et al., 2013), Rab10, Rab11, and Rab35 (Steger et al., 2016) which regulate cargo and membrane recycling from sorting endosomes back into the cycle or the endolysosomal pathway for degradation (reviewed in Taylor and Alessi, 2020). LRRK2 is also implicated in SV storage and mobilization through its phosphorylation/binding of synapsin-I (Beccano-Kelly et al., 2014; Cirnaru et al., 2014; Carrion et al., 2017; Marte et al., 2019; Marku et al., 2020). **(B)** Cartoon of a generalized postsynaptic structure showing processes in which LRRK2 has been implicated, and which are also regulated by numerous Rab GTPases (reviewed in Hausser and Schlett, 2019). While altered neurotransmitter receptor (depicted in red) composition and structural plasticity have been observed in mutant LRRK2 models (Sweet et al., 2015; Matikainen-Ankney et al., 2018), much less is known about LRRK2’s physiological role in postsynaptic processes. **(i)** Neurotransmitter receptors are removed from the plasma membrane by clathrin- and dynamin-mediated endocytosis (reviewed in Anggono and Huganir, 2012), likely involving LRRK2, Rab4, and Rab5 (Ehlers, 2000). **(ii)** As at the presynapse, VPS35 (Munsie et al., 2015), Rab10 (Glodowski et al., 2007), and Rab11 (Park et al., 2004) play a role in endosomal sorting and traffic of internalized receptors, thereby implicating LRRK2. **(iii)** LRRK2 may regulate receptor insertion into postsynaptic membranes by exocytosis *via* phosphorylation of NSF (Nishimune et al., 1998; Huang et al., 2005) and/or Rab8 (Gerges et al., 2004; Steger et al., 2016). **(iv)** LRRK2 has also been implicated in cell signaling and cytoskeletal dynamics, including altered morphology of dendritic spines (Matikainen-Ankney et al., 2018), functional interactions with ERM proteins (not depicted; Parisiadou et al., 2009) and/or protein kinase A (PKA; not depicted), which may additionally affect postsynaptic receptor expression in LRRK2 mutants (Muda et al., 2014; Parisiadou et al., 2014; Tozzi et al., 2018).

At the presynapse, LRRK2 has been linked to several proteins involved in the synaptic vesicle (SV) cycle (Figure 1A). The ATPase *N-*ethylmaleimide sensitive factor (NSF) is a central component of the cellular machinery generally employed to transfer membrane vesicles from one compartment to another, including synaptic vesicle exocytosis and endocytosis, where it catalyzes SNARE-family protein complex dissociation (Rizo and Xu, 2015). NSF was shown to co-immunoprecipitate with LRRK2 through WD40 domain interactions in brain lysate (Piccoli et al., 2011, 2014) and was subsequently identified as a LRRK2 substrate, with its phosphorylation resulting in enhanced SNARE dissociation (Belluzzi et al., 2016). Thus, LRRK2 mutations would be expected to alter vesicle recycling through NSF hyperphosphorylation.

Another player in the SV cycle is endophilin A1 (endoA), which acts early in endocytosis by inducing plasma membrane curvature (Gallop et al., 2006; Masuda et al., 2006). EndoA was reported to be phosphorylated by LRRK2 in *Drosophila*, with G2019S mutant overexpression causing increased endoA phosphorylation, and a concomitant defect in SV recycling (Matta et al., 2012). LRRK2-mediated phosphorylation of endoA has been shown to control plasma membrane association (Ambroso et al., 2014), and similar results have since been observed in mice (Arranz et al., 2015).

EndoA additionally interacts with dynamin, together regulating the fission of vesicles from the plasma membrane (Sundborger et al., 2011) and bulk endosomes (reviewed in Clayton and Cousin, 2009; Gross and von Gersdorff, 2016), and subsequently recruits synj1, which facilitates the binding of auxilin to vesicles for clathrin coat removal (Cao et al., 2017; Nguyen and Krainc, 2018; reviewed in Nguyen et al., 2019). Mutant LRRK2 disrupts the interaction between these proteins *via* hyperactive kinase activity, thereby deregulating SV trafficking (Stafa et al., 2013; Islam et al., 2016; Pan et al., 2017; Nguyen and Krainc, 2018). Interestingly, mutations in the genes encoding dynamin, auxilin, and synj1 have also been directly linked to PD (reviewed in Nguyen et al., 2019). LRRK2 may also play a role in exocytosis *via* synapsin-I, which binds and tethers SVs, thereby regulating the trafficking between the reserve pool and readily releasable pool (Fdez and Hilfiker, 2006). LRRK2 has been shown to mediate phosphorylation of synapsin-I at several sites both *in vitro* and in neurons (Beccano-Kelly et al., 2014; Cirnaru et al., 2014; Piccoli et al., 2014; Marte et al., 2019). Interestingly, the phosphorylation of Ser603 and Ser9 residues was decreased in cortical neurons from G2019S-LRRK2 knock-in mice (Beccano-Kelly et al., 2014). Neither of these residues are predicted LRRK2 phosphorylation sites; thus, the reduction suggests impaired activity of another kinase, or increased activity of a phosphatase, conferred by the G2019S mutation. A potential candidate is protein kinase A (PKA), which potentiates SV recycling *via* phosphorylation of synapsin-I on S9 (Cesca et al., 2010) and may be negatively regulated by LRRK2 (Parisiadou et al., 2014; Greggio et al., 2017). In contrast to reduced phosphorylation of Ser603 and Ser9 residues (Beccano-Kelly et al., 2014), phosphorylation of the putative LRRK2 substrate residues Thr337 and Thr339 is increased in cortical neurons expressing hG2019S-LRRK2 (Marte et al., 2019). This suggests LRRK2 phosphorylates certain synapsin-I sites, associated with altered phosphorylation at other (non-LRRK2 substrate) functional residues, and that this is altered by the G2019S mutation. Phosphorylation at tyrosine and serine sites on synapsin-I impart opposite effects on its association to actin and SVs (Cesca et al., 2010). It is noteworthy that LRRK2’s C-terminal WD40 domain was previously identified as binding synapsin-I and other SV-associated proteins (Piccoli et al., 2014), whereas recent work by the same group shows that the armadillo repeats at the N-terminus also affect LRRK2’s regulation of SV trafficking; these findings may seem contradictory at first, but the complex architecture resulting from LRRK2 dimerization may allow for both terminals to work together in shaping SV dynamics (Marku et al., 2020). Overall, altered synapsin-I phosphorylation, and other functional interactions with LRRK2, may contribute to the increased glutamate release observed in G2019S-LRRK2 neurons (Beccano-Kelly et al., 2014; Matikainen-Ankney et al., 2016; Volta et al., 2017), although an exact mechanism remains to be determined.

A further complication arises as LRRK2 may exert different effects on endo- and exo-cytosis in an activity-dependent manner. Carrion et al. (2017) showed that the LRRK2 N-terminus binds β3 Ca_V_2.1, thereby enhancing SV fusion, whereas the C-terminus binds synapsin-I and actin, which hampers exocytosis; LRRK2’s affinity to each is likely dynamically regulated by calcium concentration. Indeed, activity-dependent calcium influx influences the phosphorylation state of several SV trafficking proteins, and may, in turn, be regulated by LRRK2’s interaction with Ca_V_2.1 channels (Bedford et al., 2016). LRRK2’s potential role in regulating calcium influx is of considerable interest, given some reports that LRRK2 mutations alter mitochondrial homeostasis (Cherra et al., 2013; Bedford et al., 2016; Verma et al., 2017), and that synaptic mitochondria are a major sink for calcium buffering (see Ryan et al., 2015 for an overview of mitochondrial dysfunction in PD). Lastly, LRRK2’s association with several Rab proteins has garnered increasing attention (Shin et al., 2008; Dodson et al., 2012; MacLeod et al., 2013; Beilina et al., 2014; Cirnaru et al., 2014; Yun et al., 2015; Inoshita et al., 2017; Mir et al., 2018). Initial evidence of an interaction with Rab5b was found in GST pull-down and co-immunoprecipitation experiments (Shin et al., 2008), and more recently a large phosphoproteomic study revealed LRRK2 phosphorylates many others (Steger et al., 2016, 2017). Rab proteins are variously implicated in nearly all aspects of endosomal trafficking and recycling and additionally may provide a functional link between LRRK2 and VPS35, another PD-linked protein critical to cargo recycling in sorting endosomes (Inoshita et al., 2017; Mir et al., 2018) and with mutation-dependent effects on synaptic function (Munsie et al., 2015; Ishizu et al., 2016; Temkin et al., 2017; Cataldi et al., 2018).

Despite growing electrophysiological and morphological evidence that LRRK2 also acts postsynaptically, fewer molecular interactions have been uncovered on this side of the equation (Figure 1B). Several studies suggested LRRK2 is involved in postsynaptic receptor trafficking; these have reported altered D1 dopamine receptor distribution in the striatum of GKI mice (Migheli et al., 2013), a lack of calcium-permeable AMPA receptors in nucleus accumbens SPNs (Matikainen-Ankney et al., 2018), decreased NMDA receptor integration in synaptosomes from LRRK2 KO mice (Caesar et al., 2015), and altered NMDA/AMPA receptor ratios in hippocampal slices from hG2019S-LRRK2 transgenic mice (Sweet et al., 2015). Such trafficking could be altered due to differential phosphorylation of Rab8a (Steger et al., 2016) or NSF (Belluzzi et al., 2016), both of which are involved in AMPA subunit trafficking (Nishimune et al., 1998; Gerges et al., 2004). As mentioned previously, LRRK2 binds to, and negatively regulates, PKA (Parisiadou et al., 2014), which also modulates receptor insertion and cytoskeleton dynamics (see Greggio et al., 2017). The R1441C/G mutation disrupts the interaction between LRRK2 and PKA, causing aberrant phosphorylation of downstream proteins (Muda et al., 2014; Parisiadou et al., 2014). Lastly, a recent report found a reduction in the scaffolding protein PSD-95 within the hippocampus of hG2019S-LRRK2 transgenic mice, arguably contributing to an observed cognitive impairment (Adeosun et al., 2017). That said, PSD-95 levels were not altered in cultured cortical neurons (Beccano-Kelly et al., 2014) or striatal slices (Matikainen-Ankney et al., 2016) from GKI mice; thus, further investigation is required to determine whether discrepancies are due to age, neuronal type, and/or LRRK2 expression levels.

LRRK2’s functional interaction with α-synuclein has garnered considerable attention within the field. Although how or why they form is unknown, aberrantly phosphorylated α-syn aggregates are found variously throughout the *post-mortem* brain in synucleinopathies, including most (but not all) forms of PD (reviewed in Goedert et al., 2013; Giguère et al., 2018). Aggregated α-syn has also been found within fetal graft cells transplanted into PD patient striatum ~10 years before death; a pair of seminal reports in 2008 suggested that α-syn aggregates had either been induced in, or spread to, fetal cells by the host (Kordower et al., 2008; Li et al., 2008). This spreading pathology has led to a “prion-like” model of seeding and transmission of toxic α-syn, for which there is much evidence (Brundin and Melki, 2017); however, the priogenic spreading mechanism is hotly debated (Surmeier et al., 2017). Pathological α-syn phosphorylation and accumulation, resembling that in synucleinopathies, can be induced by application of pre-formed fibrillar α-syn (PFF) exposure in cell lines (Luk et al., 2009), neurons (Volpicelli-Daley et al., 2011), mutant *SNCA*-overexpressing mice (Luk et al., 2012b), WT mice (Luk et al., 2012a), and rats (Paumier et al., 2015). PFF-induced α-syn aggregation is increased in LRRK2 mutant scenarios (Volpicelli-Daley et al., 2016; Bieri et al., 2019; MacIsaac et al., 2020), suggesting a gain of function effect upon α-syn pathological processes. Indeed, a recent study found that LRRK2 increased α-syn propagation across multiple models in a kinase activity-dependent manner, likely *via* phosphorylation of Rab35 (Bae et al., 2018). Consistently, PFF-induced α-syn aggregation is reduced by LRRK2 germ line knock-out (MacIsaac et al., 2020) and kinase inhibition (Volpicelli-Daley et al., 2016), albeit not always robustly (Henderson et al., 2018). Interestingly, a recent study shows that LRRK2 inhibitors reduce the accumulation of phosphorylated α-syn, as well as that of oxidized dopamine products, possibly by a Rab10-dependant restoration of glucocerebrosidase activity—which may present a point of convergence with mutations in GBA1, another major PD risk factor (Ysselstein et al., 2019). Although many questions remain as to how (and where) LRRK2 and α-syn interact, these findings suggest targeting LRRK2 will have therapeutic potential beyond familial LRRK2 PD.

## Conclusions and Future Directions

The impetus for the development and characterization of preclinical models is simple; we wish to define disease phenotypes, understand their underlying mechanisms and reverse them, in our efforts to provide useful treatments, and ideally disease-modifying therapy, for patients. In this light, despite complex and deep literature, we posit that investigations of LRRK2 mutations have provided tangible advances.

Traditionally, it has been hoped that mouse models of PD would display the most obvious corollaries of end-stage PD, namely nigral cell loss, synuclein deposition, and severe motor dysfunction. Behavioural assessment has often focused on motor function, with reports on cognitive and psychiatric features beginning to emerge only in recent years. While the results of dopaminergic cell loss are well studied in toxin models that lesion the SN, LRRK2 (and α-synuclein) genetic models rarely show cell death or overt motor dysfunction. Further, it may be unreasonable, and possibly folly, to expect even quite severe alterations to dopamine transmission to manifest as an overt motor deficit in mice. A case in point is aphakia mice, which have a spontaneous mutation in the *Pitx3* transcription factor gene, that results in selective developmental loss of nigrostriatal dopamine neurons and an ~90% reduction in dorsal striatal dopamine (Hwang et al., 2003; Nunes et al., 2003; van den Munckhof et al., 2003; Smidt et al., 2004). Although blind, extensive behavioral testing showed these animals lack gross motor dysfunction but do exhibit an altered diurnal activity, manifest as hyperactivity during the day and hypoactivity during the night (when mice should be more active), in addition to cognitive impairments in tasks that require sensorimotor integration and procedural learning (Hwang et al., 2005; Ardayfio et al., 2008). That said, not developing a nigrostriatal pathway may be different from losing one. In MPTP-treated mice, depending on the treatment regimen, studies vary from reporting reduced locomotion, changes to locomotion, and even hyperactivity in the presence of severe dopamine depletion (Luchtman et al., 2009). Similarly, bilateral striatal injection of 6-OHDA to mice, resulting in ~70% loss of striatal TH, produces only modest gait alterations but does result in changes indicative of depression and anxiety (Bonito-Oliva et al., 2014). While other sensitive tests reveal a plethora of motor alterations following chemical lesions, none appear to correlate easily with the degree of nigral cell loss, or reductions in striatal dopamine (reviewed in Meredith and Kang, 2006; Meredith and Rademacher, 2011; Vingill et al., 2018).

Genetic ablation of dopamine neurons during and after development has produced similar results, where a ~90% reduction in TH neurons results in little (or no) motor dysfunction, and evidence that the remaining 10% of dopamine neurons were able to functionally compensate for the loss (Golden et al., 2013). Perhaps clearer in mice are the effects of dopamine depletion upon cognitive tasks, where mild (~25%) and moderate (~60%) depletion produces deficits in cognitive flexibility and working memory (Darvas and Palmiter, 2015), although the relative contributions of dopamine loss and cell death may differentially affect behavioral sequelae (Morgan et al., 2015).

The contributions of the dopaminergic system to cognitive behaviors in mice, and dysfunction thereof, correspond well to those observed in pre-motor and non-motor PD (Chaudhuri and Schapira, 2009; Kalia and Lang, 2016). Nuanced behavioral tasks that test such phenomena should be considered more informative of nigrostriatal dopamine function than oft-used measures of gross motor performance. A hyperactivity phenotype has been observed in several reports of LRRK2 mutant knock-in mice (Longo et al., 2014, 2017; Yue et al., 2015; Volta et al., 2017) and, when assessed, differences in higher-order functions have also been observed (e.g., differential response to conditioned social defeat stress; Matikainen-Ankney et al., 2018; Guevara et al., 2020).

The death of SNpc neurons is now widely accepted to be a consequence, rather than a cause, of PD. As in other neurodegenerative disorders, it is highly probable that neuronal dysfunction precedes neuronal death, and that a loss of appropriate synaptic pro-survival signaling may contribute to toxicity (Milnerwood and Raymond, 2010). We argue that the long prodromal period in PD must contain protracted dysfunction of neural circuits before cell loss, as it is highly unlikely any neuron will function perfectly before it expires. Thus, synaptic dysfunction is a likely neurodegenerative stressor, or at the very least a useful marker of cell stress and degenerative processes. Furthermore, the failure of dopamine replenishment to abate many non-motor symptoms and prevent disease progression, in concert with extranigral cell death in thalamic and cortical areas (reviewed in Giguère et al., 2018), together cement the widespread but often overlooked understanding that PD is a multi-system disease.

Most rodent models of PD based on LRRK2 mutations (and those in other genes) demonstrate alterations to nigral dopamine and other synaptic systems, that are likely pertinent to early disease symptoms and potentially progression. However, it remains unclear whether synaptic changes reflect pathophysiological processes that drive further dysfunction and eventual cell death, compensatory mechanisms within the circuitry, or a combination of the two. Although appropriate synaptic transmission is generally required for synapse-nuclear pro-survival signaling (Greer and Greenberg, 2008; Bading, 2013; Hagenston et al., 2020), altered synaptic glutamate transmission may underlie a particular aspect of PD pathogenesis. If α-syn is specifically enriched in excitatory terminals in the striatum, but not in TH-expressing nigral DA terminals (Maroteaux et al., 1988; Totterdell et al., 2004; Emmanouilidou and Vekrellis, 2016; Taguchi et al., 2016; Foffani and Obeso, 2018; Sulzer and Edwards, 2019), it may be that excitatory synapses are the source of pathological α-syn, which is eventually cytotoxic to nigral cells. Indeed, α-synuclein is secreted from cells (Emmanouilidou and Vekrellis, 2016), and in neurons, this is an activity-dependent process (Paillusson et al., 2013; Yamada and Iwatsubo, 2018). Thus, the increased glutamate activity seen in mutant LRRK2 mice (Beccano-Kelly et al., 2014; Matikainen-Ankney et al., 2016; Volta et al., 2017) may increase the burden of secreted α-syn, resulting in increased uptake by nigral terminals and a cascade of retrograde α-synuclein-induced pathological processes (Foffani and Obeso, 2018). Such a mechanism supports a link between prodromal striatal dysfunction in humans and a “dying-back” model of dopamine degeneration (reviewed in Tagliaferro and Burke, 2016; Foffani and Obeso, 2018).

LRRK2 kinase activity is not only enhanced by LRRK2 PD mutations, but also by mutations in VPS35 (Mir et al., 2018), another cause of clinically-typical, late-onset PD (Vilariño-Güell et al., 2011; Zimprich et al., 2011). Similarly to LRRK2, VPS35 mutations alter synaptic transmission in mouse cortical cultures (Munsie et al., 2015; Temkin et al., 2017), and dopamine release in mutant knock-in mice (Ishizu et al., 2016; Cataldi et al., 2018). Moreover, emerging evidence suggests increased LRRK2 activity in the *post-mortem* brains from people with PD (Di Maio et al., 2018), as well as in peripheral tissues (Fraser et al., 2016; Atashrazm et al., 2019). LRRK2 kinase inhibition has been shown to reverse increased synaptic transmission in GKI mice (Matikainen-Ankney et al., 2016), the impaired plasticity in transgenic G2019S-LRRK2 mice (Sweet et al., 2015), and the aforementioned PFF-induced increase in α-syn pathological phosphorylation and accumulation (Volpicelli-Daley et al., 2016). Built mostly on *in vitro* observations in a non-neuronal context, LRRK2 inhibitors are already in human trials, even though preclinical replication and mechanistic consensus are currently lacking (reviewed in Zhao and Dzamko, 2019). Should LRRK2 kinase inhibitors fail, targeted gene therapy, including the silencing of α-syn (reviewed in Brundin et al., 2015) or LRRK2 (Volta et al., 2015a,b; Zhao et al., 2017), may provide a valid alternative. Indeed, acute LRRK2 silencing by antisense oligonucleotides is tolerated by mice (Volta et al., 2015a), and is effective in reducing experimentally-induced α-syn aggregation (Zhao et al., 2017). Regardless, a much deeper understanding of LRRK2 biology and the effects of LRRK2 kinase inhibition or silencing is required to gauge clinical efficacy, guide biomarker discovery, and aid trial/patient selection.

An increasing body of evidence points towards a convergence of pathophysiological mechanisms in various forms of PD, involving both genetic and environmental etiological factors. Translating results from LRRK2 genetic models to other genetic scenarios, and more general PD pathogenic processes, may identify early points of intervention before the motor dysfunction by which PD is diagnosed. We believe an improved understanding of the neuronal function of LRRK2, including its role at the synapse, will facilitate the development of neuroprotective treatments, for not only LRRK2 but also idiopathic PD patients.

## Author Contributions

NK and AM co-wrote this review.

## Conflict of Interest

The authors declare that the research was conducted in the absence of any commercial or financial relationships that could be construed as a potential conflict of interest.

## References

[B1] Adeosun S. O. Hou X. Zheng B. Melrose H. L. Mosley T. Wang J. M. (2017). Human LRRK2 G2019S mutation represses post-synaptic protein PSD95 and causes cognitive impairment in transgenic mice. Neurobiol. Learn. Mem. 176, 139–148. 10.1016/j.nlm.2017.05.001 28487191 PMC5568523

[B2] Albanese F. Novello S. Morari M. (2019). Autophagy and LRRK2 in the aging brain. Front. Neurosci. 13:1352. 10.3389/fnins.2019.01352 31920513 PMC6928047

[B3] Ambroso M. R. Hegde B. G. Langen R. (2014). Endophilin A1 induces different membrane shapes using a conformational switch that is regulated by phosphorylation. Proc. Natl. Acad. Sci. U S A 111, 6982–6987. 10.1073/pnas.1402233111 24778241 PMC4024918

[B4] Andreae L. C. Burrone J. (2018). The role of spontaneous neurotransmission in synapse and circuit development. J. Neurosci. Res. 96, 354–359. 10.1002/jnr.24154 29034487 PMC5813191

[B5] Andreae L. C. Fredj N. B. Burrone J. (2012). Independent vesicle pools underlie different modes of release during neuronal development. J. Neurosci. 32, 1867–1874. 10.1523/JNEUROSCI.5181-11.2012 22302825 PMC6703344

[B6] Andres-Mateos E. Mejias R. Sasaki M. Li X. Lin B. M. Biskup S.. (2009). Unexpected lack of hypersensitivity in LRRK2 knock-out mice to MPTP (1-methyl-4-phenyl-1,2,3,6-tetrahydropyridine). J. Neurosci. 29, 15846–15850. 10.1523/JNEUROSCI.4357-09.2009 20016100 PMC2846613

[B7] Anggono V. Huganir R. L. (2012). Regulation of AMPA receptor trafficking and synaptic plasticity. Curr. Opin. Neurobiol. 22, 461–469. 10.1016/j.conb.2011.12.006 22217700 PMC3392447

[B8] Ardayfio P. Moon J. S. Leung K. K. A. Youn-Hwang D. Kim K. S. (2008). Impaired learning and memory in Pitx3 deficient aphakia mice: A genetic model for striatum-dependent cognitive symptoms in Parkinson’s disease. Neurobiol. Dis. 31, 406–412. 10.1016/j.nbd.2008.05.017 18573342 PMC2594011

[B9] Arranz A. M. Delbroek L. Van Kolen K. Guimaraes M. R. Mandemakers W. Daneels G.. (2015). LRRK2 functions in synaptic vesicle endocytosis through a kinase-dependent mechanism. J. Cell Sci. 128, 541–552. 10.1242/jcs.158196 25501810

[B10] Arstikaitis P. Gauthier-Campbell C. Carolina Gutierrez Herrera R. Huang K. Levinson J. N. Murphy T. H.. (2008). Paralemmin-1, a modulator of filopodia induction is required for spine maturation. Mol. Biol. Cell 19, 2026–2038. 10.1091/mbc.e07-08-0802 18287537 PMC2366842

[B11] Arstikaitis P. Gauthier-Campbell C. Huang K. El-Husseini A. Murphy T. H. (2011). Proteins that promote filopodia stability, but not number, lead to more axonal-dendritic contacts. PLoS One 6:e16998. 10.1371/journal.pone.0016998 21408225 PMC3049770

[B12] Atashrazm F. Hammond D. Perera G. Bolliger M. F. Matar E. Halliday G. M.. (2019). LRRK2-mediated Rab10 phosphorylation in immune cells from Parkinson’s disease patients. Mov. Disord. 34, 406–415. 10.1002/mds.27601 30597610

[B13] Bading H. (2013). Nuclear calcium signalling in the regulation of brain function. Nat. Rev. Neurosci. 14, 593–608. 10.1038/nrn3531 23942469

[B14] Bae E. J. Kim D. K. Kim C. Mante M. Adame A. Rockenstein E.. (2018). LRRK2 kinase regulates α-synuclein propagation *via* RAB35 phosphorylation. Nat. Commun. 9:3465. 10.1038/s41467-018-05958-z 30150626 PMC6110743

[B15] Beccano-Kelly D. A. Kuhlmann N. Tatarnikov I. Volta M. Munsie L. N. Chou P.. (2014). Synaptic function is modulated by LRRK2 and glutamate release is increased in cortical neurons of G2019S LRRK2 knock-in mice. Front. Cell. Neurosci. 8:301. 10.3389/fncel.2014.00301 25309331 PMC4176085

[B16] Beccano-Kelly D. A. Volta M. Munsie L. N. Paschall S. A. Tatarnikov I. Co K.. (2015). LRRK2 overexpression alters glutamatergic presynaptic plasticity, striatal dopamine tone, postsynaptic signal transduction, motor activity and memory. Hum. Mol. Genet. 24, 1336–1349. 10.1093/hmg/ddu543 25343991

[B17] Bedford C. Sears C. Perez-Carrion M. Piccoli G. Condliffe S. B. (2016). LRRK2 regulates voltage-gated calcium channel function. Front. Mol. Neurosci. 9:35. 10.3389/fnmol.2016.00035 27242426 PMC4876133

[B18] Beilina A. Rudenko I. N. Kaganovich A. Civiero L. Chau H. Kalia S. K.. (2014). Unbiased screen for interactors of leucine-rich repeat kinase 2 supports a common pathway for sporadic and familial Parkinson disease. Proc. Natl. Acad. Sci. U S A 111, 2626–2631. 10.1073/pnas.1318306111 24510904 PMC3932908

[B19] Belluzzi E. Gonnelli A. Cirnaru M. D. Marte A. Plotegher N. Russo I.. (2016). LRRK2 phosphorylates pre-synaptic N-ethylmaleimide sensitive fusion (NSF) protein enhancing its ATPase activity and SNARE complex disassembling rate. Mol. Neurodegener. 11:1. 10.1186/s13024-015-0066-z 26758690 PMC4711005

[B20] Bichler Z. Lim H. C. Zeng L. Tan E. K. (2013). Non-motor and motor features in LRRK2 transgenic mice. PLoS One 8:e70249. 10.1371/journal.pone.0070249 23936174 PMC3728021

[B21] Bieri G. Brahic M. Bousset L. Couthouis J. Kramer N. J. Ma R.. (2019). LRRK2 modifies α-syn pathology and spread in mouse models and human neurons. Acta Neuropathologica 137, 961–980. 10.1007/s00401-019-01995-0 30927072 PMC6531417

[B22] Biskup S. Moore D. J. Celsi F. Higashi S. West A. B. Andrabi S. A.. (2006). Localization of LRRK2 to membranous and vesicular structures in mammalian brain. Ann. Neurol. 60, 557–569. 10.1002/ana.21019 17120249

[B23] Bonito-Oliva A. Masini D. Fisone G. (2014). A mouse model of non-motor symptoms in Parkinson’s disease: focus on pharmacological interventions targeting affective dysfunctions. Front. Behav. Neurosci. 8:290. 10.3389/fnbeh.2014.00290 25221486 PMC4145811

[B24] Boyer C. Schikorski T. Stevens C. F. (1998). Comparison of hippocampal dendritic spines in culture and in brain. J. Neurosci. 18, 5294–5300. 10.1523/JNEUROSCI.18-14-05294.1998 9651212 PMC6793498

[B25] Brundin P. Melki R. (2017). Prying into the prion hypothesis for Parkinson’s disease. J. Neurosci. 37, 9808-9818. 10.1523/JNEUROSCI.1788-16.2017 29021298 PMC5637113

[B26] Brundin P. Atkin G. Lamberts J. T. (2015). Basic science breaks through: new therapeutic advances in Parkinson’s disease. Mov. Disord. 30, 1521-1527. 10.1002/mds.26332 26177603

[B27] Burguière A. De Bundel D. Valjent E. Roger J. Smolders I. Fagni L.. (2013). Combination of group I mGlu receptors antagonist with dopaminergic agonists strengthens the synaptic transmission at corticostriatal synapses in culture. Neuropharmacology 66, 151–157. 10.1016/j.neuropharm.2012.03.017 22465815

[B28] Caesar M. Felk S. Aasly J. O. Gillardon F. (2015). Changes in actin dynamics and F-actin structure both in synaptoneurosomes of LRRK2(R1441G) mutant mice and in primary human fibroblasts of LRRK2(G2019S) mutation carriers. Neuroscience 284, 311–324. 10.1016/j.neuroscience.2014.09.070 25301747

[B29] Cao M. Wu Y. Ashrafi G. McCartney A. J. Wheeler H. Bushong E. A.. (2017). Parkinson sac domain mutation in synaptojanin 1 impairs clathrin uncoating at synapses and triggers dystrophic changes in dopaminergic axons. Neuron 93, 882.e5–896.e5. 10.1016/j.neuron.2017.01.019 28231468 PMC5340420

[B30] Carrion M. D. P. Marsicano S. Daniele F. Marte A. Pischedda F. Di Cairano E.. (2017). The LRRK2 G2385R variant is a partial loss-of-function mutation that affects synaptic vesicle trafficking through altered protein interactions. Sci. Rep. 7:5377. 10.1038/s41598-017-05760-9 28710481 PMC5511190

[B31] Cataldi S. Follett J. Fox J. D. Tatarnikov I. Kadgien C. Gustavsson E. K.. (2018). Altered dopamine release and monoamine transporters in Vps35 p.D620N knock-in mice. NPJ Parkinsons Dis. 4:27. 10.1038/s41531-018-0063-3 30155515 PMC6104078

[B4007] Cesca F. Baldelli P. Valtorta F. Benfenati F. (2010). The synapsins: Key actors of synapse function and plasticity. Progress in Neurobiology 91, 313–348. 10.1016/j.pneurobio.2010.04.006 20438797

[B32] Chanaday N. L. Kavalali E. T. (2018). Optical detection of three modes of endocytosis at hippocampal synapses. Elife 7:e36097. 10.7554/eLife.36097 29683423 PMC5959719

[B33] Chartier-Harlin M.-C. Kachergus J. Roumier C. Mouroux V. Douay X. Lincoln S.. (2004). α-synuclein locus duplication as a cause of familial Parkinson’s disease. Lancet 364, 1167–1169. 10.1016/S0140-6736(04)17103-1 15451224

[B34] Chaudhuri K. R. Schapira A. H. V. (2009). Non-motor symptoms of Parkinson’s disease: dopaminergic pathophysiology and treatment. Lancet Neurol. 8, 464–474. 10.1016/S1474-4422(09)70068-7 19375664

[B35] Chen C. Y. Weng Y. H. Chien K. Y. Lin K. J. Yeh T. H. Cheng Y. P.. (2012). (G2019S) LRRK2 activates MKK4-JNK pathway and causes degeneration of SN dopaminergic neurons in a transgenic mouse model of PD. Cell Death Differ. 19, 1623–1633. 10.1038/cdd.2012.42 22539006 PMC3438494

[B36] Chen X. Xie C. Tian W. Sun L. Zheng W. Hawes S. (2020). Parkinson’s disease-related Leucine-rich repeat kinase 2 modulates nuclear morphology and genomic stability in striatal projection neurons during aging. Mol. Neurodegener. 15:12 10.1186/s13024-020-00360-0 32075681 PMC7031993

[B37] Cherra S. J. Steer E. Gusdon A. M. Kiselyov K. Chu C. T. (2013). Mutant LRRK2 elicits calcium imbalance and depletion of dendritic mitochondria in neurons. Am. J. Pathol. 182, 474–484. 10.1016/j.ajpath.2012.10.027 23231918 PMC3562730

[B38] Chesselet M. F. Richter F. (2011). Modelling of Parkinson’s disease in mice. Lancet Neurol. 10, 1108–1118. 10.1016/S1474-4422(11)70227-7 22094131

[B39] Cirnaru M. D. Marte A. Belluzzi E. Russo I. Gabrielli M. Longo F.. (2014). LRRK2 kinase activity regulates synaptic vesicle trafficking and neurotransmitter release through modulation of LRRK2 macro-molecular complex. Front. Mol. Neurosci. 7:49. 10.3389/fnmol.2014.00049 24904275 PMC4034499

[B40] Clayton E. L. Cousin M. A. (2009). The molecular physiology of activity-dependent bulk endocytosis of synaptic vesicles. J. Neurochem. 111, 901–914. 10.1111/j.1471-4159.2009.06384.x 19765184 PMC2871311

[B41] Clayton E. L. Sue N. Smillie K. J. O’Leary T. Bache N. Cheung G.. (2010). Dynamin i phosphorylation by GSK3 controls activity-dependent bulk endocytosis of synaptic vesicles. Nat. Neurosci. 13, 845–851. 10.1038/nn.2571 20526333 PMC2894011

[B42] Cresto N. Gaillard M. C. Gardier C. Gubinelli F. Diguet E. Bellet D.. (2020). The C-terminal domain of LRRK2 with the G2019S mutation is sufficient to produce neurodegeneration of dopaminergic neurons *in vivo*. Neurobiol. Dis. 134:104614. 10.1016/j.nbd.2019.104614 31605779

[B43] Dächsel J. C. Behrouz B. Yue M. Beevers J. E. Melrose H. L. Farrer M. J. (2010). A comparative study of Lrrk2 function in primary neuronal cultures. Parkinsonism Relat. Disord. 16, 650–655. 10.1016/j.parkreldis.2010.08.018 20850369 PMC3159957

[B44] Daniel G. Moore D. J. (2014). Modeling LRRK2 pathobiology in Parkinson’s disease: from yeast to rodents. Curr. Top. Behav. Neurosci. 22, 331–368. 10.1007/7854_2014_311 24850078

[B45] Darvas M. Palmiter R. D. (2015). Specific contributions of N-methyl-D-aspartate receptors in the dorsal striatum to cognitive flexibility. Neuroscience 284, 934–942. 10.1016/j.neuroscience.2014.11.011 25446363 PMC4267923

[B46] Davies P. Hinkle K. M. Sukar N. N. Sepulveda B. Mesias R. Serrano G.. (2013). Comprehensive characterization and optimization of anti-LRRK2 (leucine-rich repeat kinase 2) monoclonal antibodies. Biochem. J. 453, 101–113. 10.1042/BJ20121742 23560750 PMC3682752

[B47] de Lau L. M. Breteler M. M. (2006). The epidemiology of Parkinson’s disease. Lancet Neurol. 5, 525–535. 10.1016/S1474-4422(06)70471-9 16713924

[B48] Di Maio R. Hoffman E. K. Rocha E. M. Keeney M. T. Sanders L. H. De Miranda B. R.. (2018). LRRK2 activation in idiopathic Parkinson’s disease. Sci. Transl. Med. 10:eaar5429. 10.1126/scitranslmed.aar5429 30045977 PMC6344941

[B49] Dodson M. W. Zhang T. Jiang C. Chen S. Guo M. (2012). Roles of the *Drosophila* LRRK2 homolog in Rab7-dependent lysosomal positioning. Hum. Mol. Genet. 21, 1350–1363. 10.1093/hmg/ddr573 22171073 PMC3284123

[B50] Dranka B. P. Gifford A. Ghosh A. Zielonka J. Joseph J. Kanthasamy A. G.. (2013). Diapocynin prevents early Parkinson’s disease symptoms in the leucine-rich repeat kinase 2 (LRRK2R1441G) transgenic mouse. Neurosci. Lett. 549, 57–62. 10.1016/j.neulet.2013.05.034 23721786 PMC3729885

[B51] Ehlers M. D. (2000). Reinsertion or degradation of AMPA receptors determined by activity-dependent endocytic sorting the accumulation and half-life of postsynaptic AMPARs at synapses (O’Brien et al suggesting activity-dependent regula-tion of AMPAR degradation. More rapid los. Neuron 28, 511–525. 10.1016/s0896-6273(00)00129-x 11144360

[B52] Emmanouilidou E. Vekrellis K. (2016). Exocytosis and spreading of normal and aberrant α-synuclein. Brain Pathol. 26, 398–403. 10.1111/bpa.12373 26940375 PMC8029167

[B53] Fan Y. Howden A. J. M. Sarhan A. R. Lis P. Ito G. Martinez T. N.. (2018). Interrogating Parkinson’s disease LRRK2 kinase pathway activity by assessing Rab10 phosphorylation in human neutrophils. Biochem. J. 475, 23–44. 10.1042/BCJ20170803 29127255 PMC5748842

[B54] Fdez E. Hilfiker S. (2006). Vesicle pools and synapsins: new insights into old enigmas. Brain Cell Biol. 35, 107–115. 10.1007/s11068-007-9013-4 17957477

[B55] Foffani G. Obeso J. A. (2018). A cortical pathogenic theory of Parkinson’s disease. Neuron 99, 1116-1128. 10.1016/j.neuron.2018.07.028 30236282

[B56] Fraser K. B. Rawlins A. B. Clark R. G. Alcalay R. N. Standaert D. G. Liu N.. (2016). Ser(P)-1292 LRRK2 in urinary exosomes is elevated in idiopathic Parkinson’s disease. Mov. Disord. 31, 1543–1550. 10.1002/mds.26686 27297049 PMC5053851

[B57] Gallop J. L. Jao C. C. Kent H. M. Butler P. J. G. Evans P. R. Langen R.. (2006). Mechanism of endophilin N-BAR domain-mediated membrane curvature. EMBO J. 25, 2898–2910. 10.1038/sj.emboj.7601174 16763559 PMC1500843

[B58] Galter D. Westerlund M. Carmine A. Lindqvist E. Sydow O. Olson L. (2006). LRRK2 expression linked to dopamine-innervated areas. Ann. Neurol. 59, 714–719. 10.1002/ana.20808 16532471

[B59] Gerges N. Z. Backos D. S. Esteban J. A. (2004). Local control of AMPA receptor trafficking at the postsynaptic terminal by a small GTPase of the Rab family. J. Biol. Chem. 279, 43870–43878. 10.1074/jbc.M404982200 15297461

[B60] Giasson B. I. Duda J. E. Quinn S. M. Zhang B. Trojanowski J. Q. Lee V. M. Y. (2002). Neuronal α-synucleinopathy with severe movement disorder in mice expressing A53T human α-synuclein. Neuron 34, 521–533. 10.1016/s0896-6273(02)00682-7 12062037

[B61] Giesert F. Glasl L. Zimprich A. Ernst L. Piccoli G. Stautner C.. (2017). The pathogenic LRRK2 R1441C mutation induces specific deficits modeling the prodromal phase of Parkinson’s disease in the mouse. Neurobiol. Dis. 105, 179–193. 10.1016/j.nbd.2017.05.013 28576705

[B62] Giesert F. Hofmann A. Bürger A. Zerle J. Kloos K. Hafen U.. (2013). Expression analysis of Lrrk1, Lrrk2 and Lrrk2 splice variants in mice. PLoS One 8:e63778. 10.1371/journal.pone.0063778 23675505 PMC3651128

[B63] Giguère N. Nanni S. B. Trudeau L. E. (2018). On cell loss and selective vulnerability of neuronal populations in Parkinson’s disease. Front. Neurol. 9:455. 10.3389/fneur.2018.00455 29971039 PMC6018545

[B64] Glodowski D. R. Chen C. C.-H. Schaefer H. Grant B. D. Rongo C. (2007). RAB-10 regulates glutamate receptor recycling in a cholesterol-dependent endocytosis pathway. Mol. Biol. Cell 18, 4387–4396. 10.1091/mbc.e07-05-0486 17761527 PMC2043545

[B65] Goedert M. Spillantini M. G. Del Tredici K. Braak H. (2013). 100 years of Lewy pathology. Nat. Rev. Neurol. 9, 13–24. 10.1038/nrneurol.2012.242 23183883

[B66] Golden J. P. DeMaro J. A. Knoten A. Hoshi M. Pehek E. Johnson E. M.. (2013). Dopamine-dependent compensation maintains motor behavior in mice with developmental ablation of dopaminergic neurons. J. Neurosci. 33, 17095–17107. 10.1523/JNEUROSCI.0890-13.2013 24155314 PMC3807031

[B67] Goldman S. M. Marek K. Ottman R. Meng C. Comyns K. Chan P.. (2019). Concordance for Parkinson’s disease in twins: a 20-year update. Ann. Neurol. 85, 600–605. 10.1002/ana.25441 30786044

[B2000] Gómez-Suaga P. Rivero-Ríos M. Fdez E. Blanca Ramírez M. Ferrer I. Aiastui A.. (2014). LRRK2 delays degradative receptor trafficking by impeding late endosomal budding through decreasing Rab7 activity. Hum. Mol. Genet. 23, 6779–6796. 10.1093/hmg/ddu395 25080504

[B68] Greer P. L. Greenberg M. E. (2008). From synapse to nucleus: calcium-dependent gene transcription in the control of synapse development and function. Neuron 59, 846–860. 10.1016/j.neuron.2008.09.002 18817726

[B69] Greggio E. Bubacco L. Russo I. (2017). Cross-talk between LRRK2 and PKA: implication for Parkinson’s disease? Biochem. Soc. Trans. 45, 261–267). 10.1042/BST20160396 28202680

[B70] Gross O. P. von Gersdorff H. (2016). Recycling at synapses. eLife 5, 3–5. 10.7554/eLife.17692 PMC4927291 27352733

[B71] Guevara C. A. Matikainen-Ankney B. A. Kezunovic N. LeClair K. Conway A. P. Menard C.. (2020). LRRK2 mutation alters behavioral, synaptic and non-synaptic adaptations to acute social stress. J. Neurophysiol. 123, 2382-2389. jn.00137.2020. 10.1152/jn.00137.2020 32374202 PMC7311730

[B72] Hagenston A. Bading H. Bas-Orth C. (2020). Functional consequences of calcium-dependent synapse-to-nucleus communication: focus on transcription-dependent metabolic plasticity. Cold Spring Harbor. Perspect. Biol. 12:a035287. 10.1101/cshperspect.a035287 31570333 PMC7111253

[B73] Halliday G. M. (2009). Thalamic changes in Parkinson’s disease. Parkinsonism Relat. Disord. 15, S152-S155. 10.1016/S1353-8020(09)70804-1 20082979

[B74] Han E. B. Stevens C. F. (2009). Development regulates a switch between postand presynaptic strengthening in response to activity deprivation. Proc. Natl. Acad. Sci. U S A 106, 10817–10822. 10.1073/pnas.0903603106 19509338 PMC2705571

[B75] Harrill J. A. Chen H. Streifel K. M. Yang D. Mundy W. R. Lein P. J. (2015). Ontogeny of biochemical, morphological and functional parameters of synaptogenesis in primary cultures of rat hippocampal and cortical neurons. Mol. Brain 8:10. 10.1186/s13041-015-0099-9 25757474 PMC4339650

[B76] Hatano T. Kubo S.-I. Imai S. Maeda M. Ishikawa K. Mizuno Y.. (2007). Leucine-rich repeat kinase 2 associates with lipid rafts. Hum. Mol. Genet. 16, 678–690. 10.1093/hmg/ddm013 17341485

[B77] Hausser A. Schlett K. (2019). Coordination of AMPA receptor trafficking by Rab GTPases. Small GTPases 10, 419–432. 10.1080/21541248.2017.1337546 28628388 PMC6748377

[B78] Henderson J. M. Carpenter K. Cartwright H. Halliday G. M. (2000). Loss of thalamic intralaminar nuclei in progressive supranuclear palsy and Parkinson’s disease: clinical and therapeutic implications. Brain 123, 1410–1421. 10.1093/brain/123.7.1410 10869053

[B79] Henderson M. X. Peng C. Trojanowski J. Q. Lee V. M. Y. (2018). LRRK2 activity does not dramatically alter α-synuclein pathology in primary neurons. Acta Neuropathol. Commun. 6:45. 10.1186/s40478-018-0550-0 29855356 PMC5984465

[B80] Herzig M. C. Kolly C. Persohn E. Theil D. Schweizer T. Hafner T.. (2011). LRRK2 protein levels are determined by kinase function and are crucial for kidney and lung homeostasis in mice. Hum. Mol. Genet. 20, 4209–4223. 10.1093/hmg/ddr348 21828077 PMC3188995

[B81] Higashi S. Moore D. J. Colebrooke R. E. Biskup S. Dawson V. L. Arai H.. (2007). Expression and localization of Parkinson’s disease-associated leucine-rich repeat kinase 2 in the mouse brain. J. Neurochem. 100, 368–381. 10.1111/j.1471-4159.2006.04246.x 17101029

[B82] Hinkle K. M. Yue M. Behrouz B. Dächsel J. C. Lincoln S. J. Bowles E. E.. (2012). LRRK2 knockout mice have an intact dopaminergic system but display alterations in exploratory and motor co-ordination behaviors. Mol. Neurodegener. 7:25. 10.1186/1750-1326-7-25 22647713 PMC3441373

[B83] Huang Y. Man H. Y. Sekine-Aizawa Y. Han Y. Juluri K. Luo H.. (2005). S-nitrosylation of N-ethylmaleimide sensitive factor mediates surface expression of AMPA receptors. Neuron 46, 533–540. 10.1016/j.neuron.2005.03.028 15944123

[B84] Hwang D. Y. Ardayfio P. Kang U. J. Semina E. V. Kim K. S. (2003). Selective loss of dopaminergic neurons in the substantia nigra of Pitx3-deficient aphakia mice. Mol. Brain Res. 114, 123–131. 10.1016/s0169-328x(03)00162-1 12829322

[B85] Hwang D. Y. Fleming S. M. Ardayfio P. Moran-Gates T. Kim H. Tarazi F. I.. (2005). 3,4-Dihydroxyphenylalanine reverses the motor deficits in Pitx3-deficient Aphakia mice: Behavioral characterization of a novel genetic model of Parkinson’s disease. J. Neurosci. 25, 2132–2137. 10.1523/JNEUROSCI.3718-04.2005 15728853 PMC6726071

[B86] Inoshita T. Arano T. Hosaka Y. Meng H. Umezaki Y. Kosugi S.. (2017). Vps35 in cooperation with LRRK2 regulates synaptic vesicle endocytosis through the endosomal pathway in *Drosophila*. Hum. Mol. Genet. 26, 2933–2948. 10.1093/hmg/ddx179 28482024

[B87] Ishikawa Y. Katoh H. Negishi M. (2003). A role of Rnd1 GTPase in dendritic spine formation in hippocampal neurons. J. Neurosci. 23, 11065–11072. 10.1523/JNEUROSCI.23-35-11065.2003 14657163 PMC6741061

[B4000] Ishizu N. Yui D. Hebisawa A. Aizawa H. Cui W. Fujita Y.. (2016). Impaired striatal dopamine release in homozygous Vps35 D620N knock-in mice. Hum. Mol. Genet. 25, 4507–4517. 10.1093/hmg/ddw279 28173004

[B88] Islam M. S. Nolte H. Jacob W. Ziegler A. B. Pütz S. Grosjean Y.. (2016). Human R1441C LRRK2 regulates the synaptic vesicle proteome and phosphoproteome in a *Drosophila* model of Parkinson’s disease. Hum. Mol. Genet. 25, 5365–5382. 10.1093/hmg/ddw352 27794539 PMC6078604

[B89] Ito G. Katsemonova K. Tonelli F. Lis P. Baptista M. A. S. Shpiro N.. (2016). Phos-tag analysis of Rab10 phosphorylation by LRRK2: a powerful assay for assessing kinase function and inhibitors. Biochem. J. 473, 2671–2685. 10.1042/BCJ20160557 27474410 PMC5003698

[B90] Kalia L. V. Lang A. E. (2016). Parkinson disease in 2015: Evolving basic, pathological and clinical concepts in PD. Nat. Rev. Neurol. 12, 65–66. 10.1038/nrneurol.2015.249 26782330

[B91] Kaufman A. M. Milnerwood A. J. Sepers M. D. Coquinco A. She K. Wang L.. (2012). Opposing roles of synaptic and extrasynaptic NMDA receptor signaling in cocultured striatal and cortical neurons. J. Neurosci. 32, 3992–4003. 10.1523/JNEUROSCI.4129-11.2012 22442066 PMC6621208

[B92] Kavalali E. T. (2015). The mechanisms and functions of spontaneous neurotransmitter release. Nat. Rev. Neurosci. 16, 5–16. 10.1038/nrn3875 25524119

[B93] Keller M. F. Saad M. Bras J. Bettella F. Nicolaou N. Simón-Sánchez J.. (2012). Using genome-wide complex trait analysis to quantify “missing heritability” in Parkinson’s disease. Hum. Mol. Genet. 21, 4996–5009. 10.1093/hmg/dds335 22892372 PMC3576713

[B94] Kordower J. H. Chu Y. Hauser R. A. Freeman T. B. Olanow C. W. (2008). Lewy body-like pathology in long-term embryonic nigral transplants in Parkinson’s disease. Nat. Med. 14, 504–506. 10.1038/nm1747 18391962

[B95] Lalchandani R. R. van der Goes M.-S. Partridge J. G. Vicini S. (2013). Dopamine D2 receptors regulate collateral inhibition between striatal medium spiny neurons. J. Neurosci. 33, 14075–14086. 10.1523/JNEUROSCI.0692-13.2013 23986243 PMC3756755

[B4006] Larsen K. Madsen L. B. (2009). Sequence conservation between porcine and human LRRK2. Molecul. Biol. Rep. 36, 237–243. 10.1007/s11033-007-9172-5 17978862

[B96] Lee H. Melrose H. L. Yu M. Pare J.-F. Farrer M. J. Smith Y. (2010). Lrrk2 localization in the primate basal ganglia and thalamus: a light and electron microscopic analysis in monkeys. Exp. Neurol. 224, 438–447. 10.1016/j.expneurol.2010.05.004 20483355 PMC2906661

[B97] Lee H. Flynn R. Sharma I. Haberman E. Carling P. J. Nicholls F. J.. (2020). LRRK2 is recruited to phagosomes and co-recruits RAB8 and RAB10 in human pluripotent stem cell-derived macrophages. Stem Cell Rep. 14, 940–955. 10.1016/j.stemcr.2020.04.001 32359446 PMC7221108

[B98] Levinson J. N. El-Husseini A. (2005). Building excitatory and inhibitory synapses: balancing neuroligin partnerships. Neuron 48, 171–174. 10.1016/j.neuron.2005.09.017 16242398

[B99] Li J. Y. Englund E. Holton J. L. Soulet D. Hagell P. Lees A. J.. (2008). Lewy bodies in grafted neurons in subjects with Parkinson’s disease suggest host-to-graft disease propagation. Nat. Med. 14, 501–503. 10.1038/nm1746 18391963

[B100] Li X. Patel J. C. Wang J. Avshalumov M. V. Nicholson C. Buxbaum J. D.. (2010). Enhanced striatal dopamine transmission and motor performance with lrrk2 overexpression in mice is eliminated by familial Parkinson’s disease mutation G2019S. J. Neurosci. 30, 1788–1797. 10.1523/JNEUROSCI.5604-09.2010 20130188 PMC2858426

[B101] Li Y. Liu W. Oo T. F. Wang L. Tang Y. Jackson-Lewis V.. (2009). Mutant LRRK2 R1441G BAC transgenic mice recapitulate cardinal features of Parkinson’s disease. Nat. Neurosci. 12, 826–828. 10.1038/nn.2349 19503083 PMC2845930

[B102] Lim J. Bang Y. Choi J. H. Han A. Kwon M. S. Liu K. H.. (2018). LRRK2 G2019S induces anxiety/depression-like behavior before the onset of motor dysfunction with 5-HT 1A receptor upregulation in mice. J. Neurosci. 38, 1611–1621. 10.1523/JNEUROSCI.4051-15.2017 29305532 PMC6705874

[B103] Lin X. Parisiadou L. Gu X.-L. Wang L. Shim H. Sun L.. (2009). Leucine-rich repeat kinase 2 regulates the progression of neuropathology induced by Parkinson’s disease-related mutant α-synuclein. Neuron 64, 807–827. 10.1016/j.neuron.2009.11.006 20064389 PMC2807409

[B106] Liu Y. Beyer A. Aebersold R. (2016). On the dependency of cellular protein levels on mRNA abundance. Cell 165, 535–550. 10.1016/j.cell.2016.03.014 27104977

[B105] Liu H. F. Lu S. Ho P. W. L. Tse H. M. Pang S. Y. Y. Kung M. H. W.. (2014). LRRK2 R1441G mice are more liable to dopamine depletion and locomotor inactivity. Ann. Clin. Transl. Neurol. 1, 199–208. 10.1002/acn3.45 25356398 PMC4184549

[B104] Liu G. Sgobio C. Gu X. Sun L. Lin X. Yu J.. (2015). Selective expression of Parkinson’s disease-related Leucine-rich repeat kinase 2 G2019S missense mutation in midbrain dopaminergic neurons impairs dopamine release and dopaminergic gene expression. Hum. Mol. Genet. 24, 5299–5312. 10.1093/hmg/ddv249 26123485 PMC4550828

[B107] Liu Z. Wang X. Yu Y. Li X. Wang T. Jiang H.. (2008). A *Drosophila* model for LRRK2-linked parkinsonism. Proc. Natl. Acad. Sci. U S A 105, 2693–2698. 10.1073/pnas.0708452105 18258746 PMC2268198

[B108] Longo F. Mercatelli D. Novello S. Arcuri L. Brugnoli A. Vincenzi F.. (2017). Age-dependent dopamine transporter dysfunction and Serine129 phospho-α-synuclein overload in G2019S LRRK2 mice. Acta Neuropathol. Commun. 5:22. 10.1186/s40478-017-0426-8 28292328 PMC5351259

[B109] Longo F. Russo I. Shimshek D. R. Greggio E. Morari M. (2014). Genetic and pharmacological evidence that G2019S LRRK2 confers a hyperkinetic phenotype, resistant to motor decline associated with aging. Neurobiol. Dis. 71, 62–73. 10.1016/j.nbd.2014.07.013 25107341 PMC4194318

[B110] Luchtman D. W. Shao D. Song C. (2009). Behavior, neurotransmitters and inflammation in three regimens of the MPTP mouse model of Parkinson’s disease. Physiol. Behav. 98, 130–138. 10.1016/j.physbeh.2009.04.021 19410592

[B112] Luk K. C. Kehm V. Carroll J. Zhang B. O’Brien P. Trojanowski J. Q.. (2012a). Pathological α-synuclein transmission initiates parkinson-like neurodegeneration in nontransgenic mice. Science 338, 949–953. 10.1126/science.1227157 23161999 PMC3552321

[B111] Luk K. C. Kehm V. M. Zhang B. O’Brien P. Trojanowski J. Q. Lee V. M. Y. (2012b). Intracerebral inoculation of pathological α-synuclein initiates a rapidly progressive neurodegenerative α-synucleinopathy in mice. J. Exp. Med. 209, 975–988. 10.1084/jem.20112457 22508839 PMC3348112

[B113] Luk K. C. Song C. O’Brien P. Stieber A. Branch J. R. Brunden K. R.. (2009). Exogenous α-synuclein fibrils seed the formation of Lewy body-like intracellular inclusions in cultured cells. Proc. Natl. Acad. Sci. U S A 106, 20051–20056. 10.1073/pnas.0908005106 19892735 PMC2785290

[B114] Maas J. W. Jr. Yang J. Edwards R. H. (2017). Endogenous leucine-rich repeat kinase 2 slows synaptic vesicle recycling in striatal neurons. Front. Synaptic Neurosci. 9:5. 10.3389/fnsyn.2017.00005 28280464 PMC5322269

[B115] MacDonald V. Halliday G. M. (2002). Selective loss of pyramidal neurons in the pre-supplementary motor cortex in Parkinson’s disease. Mov. Disord. 17, 1166–1173. 10.1002/mds.10258 12465053

[B116] MacIsaac S. Quevedo-Melo T. Zhang Y. Volta M. Farrer M. J. Milnerwood A. J. (2020). Neuron-autonomous susceptibility to induced synuclein aggregation is exacerbated by endogenous *Lrrk2* mutations and ameliorated by *Lrrk2* genetic knock-out. Brain Commun. 2:fcz052. 10.1093/braincomms/fcz052 32510053 PMC7273240

[B118] MacLeod D. Dowman J. Hammond R. Leete T. Inoue K. Abeliovich A. (2006). The familial Parkinsonism gene LRRK2 regulates neurite process morphology. Neuron 52, 587–593. 10.1016/j.neuron.2006.10.008 17114044

[B117] MacLeod D. A. Rhinn H. Kuwahara T. Zolin A. Di Paolo G. MacCabe B. D.. (2013). RAB7L1 interacts with LRRK2 to modify intraneuronal protein sorting and Parkinson’s disease risk. Neuron 77, 425–439. 10.1016/j.neuron.2012.11.033 23395371 PMC3646583

[B4005] Maekawa T. Kubo M. Yokoyama I. Ohta E. Obata F. (2010). Age-dependent and cell-population-restricted LRRK2 expression in normal mouse spleen. Biochem. Biophys. Res. Comm. 392, 431–435. 10.1016/j.bbrc.2010.01.041 20079710

[B119] Mandemakers W. Snellinx A. O’Neill M. J. de Strooper B. (2012). LRRK2 expression is enriched in the striosomal compartment of mouse striatum. Neurobiol. Dis. 48, 582–593. 10.1016/j.nbd.2012.07.017 22850484

[B120] Manning G. Whyte D. B. Martinez R. Hunter T. Sudarsanam S. (2002). The protein kinase complement of the human genome. Science 298, 1912–1934. 10.1126/science.1075762 12471243

[B121] Marku A. Carrion M. D. P. Pischedda F. Marte A. Casiraghi Z. Marciani P.. (2020). The LRRK2 N-terminal domain influences vesicle trafficking: impact of the E193K variant. Sci. Rep. 10:3799. 10.1038/s41598-020-60834-5 32123243 PMC7052203

[B4003] Marín I. (2008). Ancient origin of the Parkinson disease gene LRRK2. J. molecul. evol. 67, 41–50. 10.1007/s00239-008-9122-4 18523712

[B122] Maroteaux L. Campanelli J. T. Scheller R. H. (1988). Synuclein: a neuron-specific protein localized to the nucleus and presynaptic nerve terminal. J. Neurosci. 8, 2804–2815. 10.1523/JNEUROSCI.08-08-02804.1988 3411354 PMC6569395

[B123] Marte A. Russo I. Rebosio C. Valente P. Belluzzi E. Pischedda F.. (2019). Leucine-rich repeat kinase 2 phosphorylation on synapsin I regulates glutamate release at pre-synaptic sites. J. Neurochem. 150, 264–281. 10.1111/jnc.14778 31148170

[B124] Masuda M. Takeda S. Sone M. Ohki T. Mori H. Kamioka Y.. (2006). Endophilin BAR domain drives membrane curvature by two newly identified structure-based mechanisms. EMBO J. 25, 2889–2897. 10.1038/sj.emboj.7601176 16763557 PMC1500852

[B125] Matikainen-Ankney B. A. Kezunovic N. Menard C. Flanigan M. Zhong Y. Russo S. J.. (2018). Parkinson’s Disease-linked LRRK2–G2019S mutation alters synaptic plasticity and promotes resilience to chronic social stress in young adulthood. J. Neurosci. 38, 9700–9711. 10.1523/JNEUROSCI.1457-18.2018 30249796 PMC6222060

[B126] Matikainen-Ankney B. A. Kezunovic N. Mesias R. E. Tian Y. Williams F. M. Huntley G. W.. (2016). Altered development of synapse structure and function in striatum caused by Parkinson’s disease-linked LRRK2–G2019S mutation. J. Neurosci. 36, 7128–7141. 10.1523/JNEUROSCI.3314-15.2016 27383589 PMC4938860

[B127] Matta S. Van Kolen K. da Cunha R. van den Bogaart G. Mandemakers W. Miskiewicz K.. (2012). LRRK2 controls an EndoA phosphorylation cycle in synaptic endocytosis. Neuron 75, 1008–1021. 10.1016/j.neuron.2012.08.022 22998870

[B128] Meixner A. Boldt K. Van Troys M. Askenazi M. Gloeckner C. J. Bauer M.. (2011). A QUICK screen for Lrrk2 interaction partners—leucine-rich repeat kinase 2 is involved in actin cytoskeleton dynamics. Mol. Cell. Proteom. 10:M110. 10.1074/mcp.M110.001172 20876399 PMC3013447

[B129] Melrose H. L. Dächsel J. C. Behrouz B. Lincoln S. J. Yue M. Hinkle K. M.. (2010). Impaired dopaminergic neurotransmission and microtubule-associated protein tau alterations in human LRRK2 transgenic mice. Neurobiol. Dis. 40, 503–517. 10.1016/j.nbd.2010.07.010 20659558 PMC2955774

[B130] Melrose H. L. Lincoln S. Tyndall G. Dickson D. Farrer M. (2006). Anatomical localization of leucine-rich repeat kinase 2 in mouse brain. Neuroscience 139, 791–794. 10.1016/j.neuroscience.2006.01.017 16504409

[B131] Mensah P. L. (1982). An electron microscopical study of neuronal cell clustering in postnatal mouse striatum, with special emphasis on neuronal cell death. Anat. Embryol. 164, 387–401. 10.1007/BF00315760 7137586

[B132] Mercatelli D. Bolognesi P. Frassineti M. Pisanò C. A. Longo F. Shimshek D. R.. (2019). Leucine-rich repeat kinase 2 (LRRK2) inhibitors differentially modulate glutamate release and Serine935 LRRK2 phosphorylation in striatal and cerebrocortical synaptosomes. Pharmacol. Res. Perspect. 7:e00484. 10.1002/prp2.484 31149340 PMC6536420

[B133] Meredith G. E. Kang U. J. (2006). Behavioral models of Parkinsons disease in rodents: a new look at an old problem. Mov. Disord. 21, 1595–1606. 10.1002/mds.21010 16830310

[B134] Meredith G. E. Rademacher D. J. (2011). MPTP mouse models of Parkinson’s disease: an update. J. Parkinsons Dis. 1, 19–33. 10.3233/JPD-2011-11023 23275799 PMC3530193

[B135] Migheli R. Del Giudice M. G. Spissu Y. Sanna G. Xiong Y. Dawson T. M.. (2013). LRRK2 affects vesicle trafficking, neurotransmitter extracellular level and membrane receptor localization. PLoS One 8:e77198. 10.1371/journal.pone.0077198 24167564 PMC3805556

[B136] Milnerwood A. J. Raymond L. A. (2010). Early synaptic pathophysiology in neurodegeneration: insights from Huntington’s disease. Trends Neurosci. 33, 513–523. 10.1016/j.tins.2010.08.002 20850189

[B137] Milnerwood A. J. Kaufman A. M. Sepers M. D. Gladding C. M. Zhang L. Wang L.. (2012). Mitigation of augmented extrasynaptic NMDAR signaling and apoptosis in cortico-striatal co-cultures from Huntington’s disease mice. Neurobiol. Dis. 48, 40–51. 10.1016/j.nbd.2012.05.013 22668780

[B138] Mir R. Tonelli F. Lis P. Macartney T. Polinski N. K. Martinez T. N.. (2018). The Parkinson’s disease VPS35[D620N] mutation enhances LRRK2-mediated Rab protein phosphorylation in mouse and human. Biochem. J. 475, 1861–1883. 10.1042/BCJ20180248 29743203 PMC5989534

[B4008] Morgan R. G. Gibbs J. T. Melief E. J. Postupna N. O. Sherfield E. E. Wilson A.. (2015). Relative contributions of severe dopaminergic neuron ablation and dopamine depletion to cognitive impairment. Experim. neurol. 271, 205–214. 10.1016/j.expneurol.2015.06.0138 26079646 PMC4586402

[B139] Mozhayeva M. G. Sara Y. Liu X. Kavalali E. T. (2002). Development of vesicle pools during maturation of hippocampal synapses. J. Neurosci. 22, 654–665. 10.1523/JNEUROSCI.22-03-00654.2002 11826095 PMC6758530

[B140] Muda K. Bertinetti D. Gesellchen F. Hermann J. S. Von Zweydorf F. Geerlof A.. (2014). Parkinson-related LRRK2 mutation R1441C/G/H impairs PKA phosphorylation of LRRK2 and disrupts its interaction with 14–3-3. Proc. Natl. Acad. Sci. U S A 111, E34–E43. 10.1073/pnas.1312701111 24351927 PMC3890784

[B141] Munsie L. N. Milnerwood A. J. Seibler P. Beccano-Kelly D. A. Tatarnikov I. Khinda J.. (2015). Retromer-dependent neurotransmitter receptor trafficking to synapses is altered by the Parkinson’s disease VPS35 mutation p.D620N. Hum. Mol. Genet. 24, 1691–1703. 10.1093/hmg/ddu582 25416282

[B142] Nguyen M. Krainc D. (2018). LRRK2 phosphorylation of auxilin mediates synaptic defects in dopaminergic neurons from patients with Parkinson’s disease. Proc. Natl. Acad. Sci. U S A 115, 5576–5581. 10.1073/pnas.1717590115 29735704 PMC6003526

[B143] Nguyen M. Wong Y. C. Ysselstein D. Severino A. Krainc D. (2019). Synaptic, mitochondrial, and lysosomal dysfunction in Parkinson’s disease. Trends Neurosci. 42, 140–149. 10.1016/j.tins.2018.11.001 30509690 PMC6452863

[B144] Nishimune A. Isaac J. T. R. Molnar E. Noel J. Nash S. R. Tagaya M.. (1998). NSF binding to GluR2 regulates synaptic transmission. Neuron 21, 87–97. 10.1016/s0896-6273(00)80517-6 9697854

[B145] Novello S. Arcuri L. Dovero S. Dutheil N. Shimshek D. R. Bezard E.. (2018). G2019S LRRK2 mutation facilitates α-synuclein neuropathology in aged mice. Neurobiol. Dis. 120, 21–33. 10.1016/j.nbd.2018.08.018 30172844

[B146] Nunes I. Tovmasian L. T. Silva R. M. Burke R. E. Goff S. P. (2003). Pitx3 is required for development of substantia nigra dopaminergic neurons. Proc. Natl. Acad. Sci. U S A 100, 4245–4250. 10.1073/pnas.0230529100 12655058 PMC153078

[B147] Oertel W. H. (2017). Recent advances in treating Parkinson’s disease. F1000Res. 6:260. 10.12688/f1000research.10100.1 28357055 PMC5357034

[B148] Paillusson S. Clairembault T. Biraud M. Neunlist M. Derkinderen P. (2013). Activity-dependent secretion of α-synuclein by enteric neurons. J. Neurochem. 125, 512–517. 10.1111/jnc.12131 23278133

[B149] Paisan-Ruiz C. Jain S. Evans E. W. Gilks W. P. Simón J. van der Brug M.. (2004). Cloning of the gene containing mutations that cause PARK8-linked Parkinson’s disease. Neuron 44, 595–600. 10.1016/j.neuron.2004.10.023 15541308

[B150] Pan P. Y. Li X. Wang J. Powell J. Wang Q. Zhang Y.. (2017). Parkinson’s disease-associated LRRK2 hyperactive kinase mutant disrupts synaptic vesicle trafficking in ventral midbrain neurons. J. Neurosci. 37, 11366–11376. 10.1523/JNEUROSCI.0964-17.2017 29054882 PMC5700420

[B151] Papa M. Bundman M. C. Greenberger V. Segal M. (1995). Morphological analysis of dendritic spine development in primary cultures of hippocampal neurons. J. Neurosci. 15, 1–11. 10.1523/JNEUROSCI.15-01-00001.1995 7823120 PMC6578316

[B152] Parisiadou L. Xie C. Cho H. J. Lin X. Gu X.-L. Long C.-X.. (2009). Phosphorylation of ezrin/radixin/moesin proteins by LRRK2 promotes the rearrangement of actin cytoskeleton in neuronal morphogenesis. J. Neurosci. 29, 13971–13980. 10.1523/JNEUROSCI.3799-09.2009 19890007 PMC2807632

[B153] Parisiadou L. Yu J. Sgobio C. Xie C. Liu G. Sun L.. (2014). LRRK2 regulates synaptogenesis and dopamine receptor activation through modulation of PKA activity. Nat. Neurosci. 17, 367–376. 10.1038/nn.3636 24464040 PMC3989289

[B154] Park M. Penick E. C. Edwards J. G. Kauer J. A. Ehlers M. D. (2004). Recycling endosomes supply AMPA receptors for LTP. Science 305, 1972–1975. 10.1126/science.1102026 15448273

[B155] Parkinson J. (2002). An essay on the shaking palsy. Neuropsychiatry Classics 14, 223–236. 10.1176/jnp.14.2.223 11983801

[B156] Paumier K. L. Luk K. C. Manfredsson F. P. Kanaan N. M. Lipton J. W. Collier T. J.. (2015). Intrastriatal injection of pre-formed mouse α-synuclein fibrils into rats triggers α-synuclein pathology and bilateral nigrostriatal degeneration. Neurobiol. Dis. 82, 185–199. 10.1016/j.nbd.2015.06.003 26093169 PMC4640952

[B157] Pellegrini L. Wetzel A. Grannó S. Heaton G. Harvey K. (2017). Back to the tubule: microtubule dynamics in Parkinson’s disease. Cell. Mol. Life Sci. 74, 409–434. 10.1007/s00018-016-2351-6 27600680 PMC5241350

[B158] Penrod R. D. Campagna J. Panneck T. Preese L. Lanier L. M. (2015). The presence of cortical neurons in striatal-cortical co-cultures alters the effects of dopamine and BDNF on medium spiny neuron dendritic development. Front. Cell. Neurosci. 9:269. 10.3389/fncel.2015.00269 26257605 PMC4507052

[B159] Piccoli G. Condliffe S. B. Bauer M. Giesert F. Boldt K. De Astis S.. (2011). LRRK2 controls synaptic vesicle storage and mobilization within the recycling pool. J. Neurosci. 31, 2225–2237. 10.1523/JNEUROSCI.3730-10.2011 21307259 PMC6633036

[B160] Piccoli G. Onofri F. Cirnaru M. D. Kaiser C. J. O. Jagtap P. Kastenmüller A.. (2014). Leucine-rich repeat kinase 2 binds to neuronal vesicles through protein interactions mediated by its C-terminal WD40 domain. Mol. Cell. Biol. 34, 2147–2161. 10.1128/MCB.00914-13 24687852 PMC4054300

[B161] Polymeropoulos M. H. Lavedan C. Hollmann M. Glutamate I. Silva A. J. Stevens C. F.. (1997). Mutation in the α-synuclein gene identified in families with Parkinson’s disease. Science 276, 2045–2048. 10.1126/science.276.5321.2045 9197268

[B162] Rajput A. Dickson D. W. Robinson C. A. Ross O. A. Dachsel J. C. Lincoln S. J.. (2006). Parkinsonism, Lrrk2 G2019S, and tau neuropathology. Neurology 67, 1506–1508. 10.1212/01.wnl.0000240220.33950.0c 17060589

[B163] Ramonet D. Daher J. P. L. Lin B. M. Stafa K. Kim J. Banerjee R.. (2011). Dopaminergic neuronal loss, reduced neurite complexity and autophagic abnormalities in transgenic mice expressing G2019S mutant LRRK2. PLoS One 6:e18568. 10.1371/journal.pone.0018568 21494637 PMC3071839

[B164] Randall F. E. Garcia-Munoz M. Vickers C. Schock S. C. Staines W. A. Arbuthnott G. W. (2011). The corticostriatal system in dissociated cell culture. Front. Syst. Neurosci. 5:52. 10.3389/fnsys.2011.00052 21743806 PMC3127227

[B165] Rizo J. Xu J. (2015). The synaptic vesicle release machinery. Annu. Rev. Biophys. 44, 339–367. 10.1146/annurev-biophys-060414-034057 26098518

[B1000] Rivero-Ríos P. Romo-Lozano M. Madero-Pérez J. Thomas A. P. Biosa A. Greggio E.. (2019). The G2019S variant of leucine-rich repeat kinase 2 (LRRK2) alters endolysosomal trafficking by impairing the function of the GTPase RAB8A. The Journal of Biological Chemistry. 294, 4738–4758. 10.1074/jbc.RA118.005008 30709905 PMC6442034

[B3000] Rivero-Ríos P. Romo-Lozano M. Fasiczka R. Naaldijk Y. Aiastui S. (2020). LRRK2-Related Parkinson’s disease due to altered endolysosomal biology with variable lewy body pathology: A hypothesis. Front. Neurosci.. 14:556. 10.3389/fnins.2020.00556 32581693 PMC7287096

[B166] Ryan B. J. Hoek S. Fon E. A. Wade-Martins R. (2015). Mitochondrial dysfunction and mitophagy in Parkinson’s: from familial to sporadic disease. Trends Biochem. Sci. 40, 200–210. 10.1016/j.tibs.2015.02.003 25757399

[B167] Sala C. Segal M. (2014). Dendritic spines: the locus of structural and functional plasticity. Physiol. Rev. 94, 141–188. 10.1152/physrev.00012.2013 24382885

[B168] Schreij A. M. Chaineau M. Ruan W. Lin S. Barker P. A. Fon E. A.. (2015). LRRK 2 localizes to endosomes and interacts with clathrin-light chains to limit Rac1 activation. EMBO Rep. 16, 79–86. 10.15252/embr.201438714 25427558 PMC4304731

[B169] Segal M. Greenberger V. Korkotian E. (2003). Formation of dendritic spines in cultured striatal neurons depends on excitatory afferent activity. Eur. J. Neurosci. 17, 2573–2585. 10.1046/j.1460-9568.2003.02696.x 12823464

[B170] Sepulveda B. Mesias R. Li X. Yue Z. Benson D. L. (2013). Short- and long-term effects of LRRK2 on axon and dendrite growth. PLoS One 8:e61986. 10.1371/journal.pone.0061986 23646112 PMC3640004

[B171] Shehadeh J. Fernandes H. B. Zeron Mullins M. M. Graham R. K. Leavitt B. R. Hayden M. R.. (2006). Striatal neuronal apoptosis is preferentially enhanced by NMDA receptor activation in YAC transgenic mouse model of Huntington disease. Neurobiol. Dis. 21, 392–403. 10.1016/j.nbd.2005.08.001 16165367

[B172] Shi M. M. Shi C. H. Xu Y. M. (2017). Rab GTPases: the key players in the molecular pathway of Parkinson’s disease. Front. Cell. Neurosci. 11:81. 10.3389/fncel.2017.00081 28400718 PMC5369176

[B173] Shin N. Jeong H. Kwon J. Heo H. Y. Kwon J. J. Yun H. J.. (2008). LRRK2 regulates synaptic vesicle endocytosis. Exp. Cell Res. 314, 2055–2065. 10.1016/j.yexcr.2008.02.015 18445495

[B174] Simón-Sánchez J. Herranz-Pérez V. Olucha-Bordonau F. Pérez-Tur J. (2006). LRRK2 is expressed in areas affected by Parkinson’s disease in the adult mouse brain. Eur. J. Neurosci. 23, 659–666. 10.1111/j.1460-9568.2006.04616.x 16487147

[B175] Singleton A. B. Farrer M. Johnson J. Singleton A. Hague S. Kachergus J.. (2003). α-synuclein locus triplication causes Parkinson’s disease. Science 302:841. 10.1126/science.1090278 14593171

[B176] Smidt M. P. Smits S. M. Burbach J. P. H. (2004). Homeobox gene Pitx3 and its role in the development of dopamine neurons of the substantia nigra. Cell Tissue Res. 318, 35–43. 10.1007/s00441-004-0943-1 15300495

[B177] Soukup S. F. Kuenen S. Vanhauwaert R. Manetsberger J. Hernández-Díaz S. Swerts J.. (2016). A LRRK2-dependent endophilinA phosphoswitch is critical for macroautophagy at presynaptic terminals. Neuron 92, 829–844. 10.1016/j.neuron.2016.09.037 27720484

[B178] Stafa K. Tsika E. Moser R. Musso A. Glauser L. Jones A.. (2013). Functional interaction of Parkinson’s disease-associated LRRK2 with members of the dynamin GTPase superfamily. Hum. Mol. Genet. 23, 2055–2077. 10.1093/hmg/ddt600 24282027 PMC3959816

[B179] Steger M. Diez F. Dhekne H. S. Lis P. Nirujogi R. S. Karayel O.. (2017). Systematic proteomic analysis of LRRK2-mediated rab GTPase phosphorylation establishes a connection to ciliogenesis. Elife 6:e31012. 10.7554/eLife.31012 29125462 PMC5695910

[B180] Steger M. Tonelli F. Ito G. Davies P. Trost M. Vetter M.. (2016). Phosphoproteomics reveals that Parkinson’s disease kinase LRRK2 regulates a subset of Rab GTPases. Elife 5:e12813. 10.7554/eLife.12813 26824392 PMC4769169

[B181] Sulzer D. Edwards R. H. (2019). The physiological role of α-synuclein and its relationship to Parkinson’s disease. J. Neurochem. 150, 475–486. 10.1111/jnc.14810 31269263 PMC6707892

[B182] Sundborger A. Soderblom C. Vorontsova O. Evergren E. Hinshaw J. E. Shupliakov O. (2011). An endophilin-dynamin complex promotes budding of clathrin-coated vesicles during synaptic vesicle recycling. J. Cell Sci. 124, 133–143. 10.1242/jcs.072686 21172823 PMC3001412

[B183] Surmeier D. J. Obeso J. A. Halliday G. M. (2017). Selective neuronal vulnerability in Parkinson disease. Nat. Rev. Neurosci. 18, 101–113. 10.1038/nrn.2016.178 28104909 PMC5564322

[B184] Sweet E. S. Saunier-Rebori B. Yue Z. Blitzer R. D. (2015). The Parkinson’s disease-associated mutation LRRK2–G2019S impairs synaptic plasticity in mouse hippocampus. J. Neurosci. 35, 11190–11195. 10.1523/JNEUROSCI.0040-15.2015 26269629 PMC4532754

[B185] Tagliaferro P. Burke R. E. (2016). Retrograde axonal degeneration in parkinson disease. J Parkinsons Dis. 6, 1–15. 10.3233/jpd-150769 27003783 PMC4927911

[B186] Taguchi K. Watanabe Y. Tsujimura A. Tanaka M. (2016). Brain region-dependent differential expression of α-synuclein. J. Comp. Neurol. 524, 1236–1258. 10.1002/cne.23901 26358191

[B187] Taguchi K. Watanabe Y. Tsujimura A. Tanaka M. (2019). Expression of α-synuclein is regulated in a neuronal cell type-dependent manner. Anat. Sci. Int. 94, 11–22. 10.1007/s12565-018-0464-8 30362073 PMC6315015

[B188] Takahashi H. Sekino Y. Tanaka S. Mizui T. Kishi S. Shirao T. (2003). Drebrin-dependent actin clustering in dendritic filopodia governs synaptic targeting of postsynaptic density-95 and dendritic spine morphogenesis. J. Neurosci. 23, 6586–6595. 10.1523/JNEUROSCI.23-16-06586.2003 12878700 PMC6740629

[B189] Taylor M. Alessi D. R. (2020). Advances in elucidating the function of leucine-rich repeat protein kinase-2 in normal cells and Parkinson’s disease. Curr. Opin. Cell Biol. 63, 102–113. 10.1016/j.ceb.2020.01.001 32036294 PMC7262585

[B5000] Temkin P. Morishita W. Goswami D. Arendt K. Chen L. Malenka R. (2017). The retromer supports AMPA receptor trafficking during LTP. Neuron. 94, 74–82. 10.1016/j.neuron.2017.03.020 28384478

[B190] Tian X. Kai L. Hockberger P. E. Wokosin D. L. Surmeier D. J. (2010). MEF-2 regulates activity-dependent spine loss in striatopallidal medium spiny neurons. Mol. Cell. Neurosci. 44, 94–108. 10.1016/j.mcn.2010.01.012 20197093 PMC2878643

[B191] Tong Y. Pisani A. Martella G. Karouani M. Yamaguchi H. Pothos E. N.. (2009). R1441C mutation in LRRK2 impairs dopaminergic neurotransmission in mice. Proc. Natl. Acad. Sci. U S A 106, 14622–14627. 10.1073/pnas.0906334106 19667187 PMC2732807

[B192] Tong Y. Yamaguchi H. Giaime E. Boyle S. Kopan R. Kelleher R. J.. (2010). Loss of leucine-rich repeat kinase 2 causes impairment of protein degradation pathways, accumulation of α-synuclein and apoptotic cell death in aged mice. Proc. Natl. Acad. Sci. U S A 107, 9879–9884. 10.1073/pnas.1004676107 20457918 PMC2906862

[B193] Totterdell S. Hanger D. Meredith G. E. (2004). The ultrastructural distribution of α-synuclein-like protein in normal mouse brain. Brain Res. 1004, 61–72. 10.1016/j.brainres.2003.10.072 15033420

[B194] Tozzi A. Tantucci M. Marchi S. Mazzocchetti P. Morari M. Pinton P.. (2018). Dopamine D2 receptor-mediated neuroprotection in a G2019S Lrrk2 genetic model of Parkinson’s disease. Cell Death Dis. 9:204. 10.1038/s41419-017-0221-2 29434188 PMC5833812

[B195] Ujiie S. Hatano T. Kubo S. I. Imai S. Sato S. Uchihara T.. (2012). LRRK2 I2020T mutation is associated with tau pathology. Parkinsonism Relat. Disord. 18, 819–823. 10.1016/j.parkreldis.2012.03.024 22525366

[B196] van den Munckhof P. Luk K. C. Ste-Marie L. Montgomery J. Blanchet P. J. Sadikot A. F.. (2003). Pitx3 is required for motor activity and for survival of a subset of midbrain dopaminergic neurons. Development 130, 2535–2542. 10.1242/dev.00464 12702666

[B197] Verma M. Callio J. Anthony Otero P. Sekler I. Wills Z. P. Chu C. T. (2017). Mitochondrial calcium dysregulation contributes to dendrite degeneration mediated by PD/LBD-Associated LRRK2 mutants. J. Neurosci. 37, 11151–11165. 10.1523/JNEUROSCI.3791-16.2017 29038245 PMC5688524

[B198] Vilariño-Güell C. Wider C. Ross O. A. Dachsel J. C. Kachergus J. M. Lincoln S. J.. (2011). VPS35 mutations in parkinson disease. Am. J. Hum. Genet. 89, 162–167. 10.1016/j.ajhg.2011.06.001 21763482 PMC3135796

[B199] Vingill S. Connor-Robson N. Wade-Martins R. (2018). Are rodent models of Parkinson’s disease behaving as they should? Behav. Brain Res. 352, 133–141. 10.1016/j.bbr.2017.10.021 29074404

[B200] Volpicelli-Daley L. A. Abdelmotilib H. Liu Z. Stoyka L. Daher J. P. L. Milnerwood A. J.. (2016). G2019s-LRRK2 expression augments α-synuclein sequestration into inclusions in neurons. J. Neurosci. 36, 7415–7427. 10.1523/JNEUROSCI.3642-15.2016 27413152 PMC4945663

[B201] Volpicelli-Daley L. A. Luk K. C. Patel T. P. Tanik S. A. Riddle D. M. Stieber A.. (2011). Exogenous α-synuclein fibrils induce lewy body pathology leading to synaptic dysfunction and neuron death. Neuron 72, 57–71. 10.1016/j.neuron.2011.08.033 21982369 PMC3204802

[B202] Volta M. Beccano-Kelly D. A. Paschall S. A. Cataldi S. Macisaac S. E. Kuhlmann N.. (2017). Initial elevations in glutamate and dopamine neurotransmission decline with age, as does exploratory behavior, in LRRK2 G2019S knock-in mice. ELife 6:e28377. 10.7554/eLife.28377 28930069 PMC5633343

[B203] Volta M. Cataldi S. Beccano-Kelly D. Munsie L. Tatarnikov I. Chou P.. (2015a). Chronic and acute LRRK2 silencing has no long-term behavioral effects, whereas wild-type and mutant LRRK2 overexpression induce motor and cognitive deficits and altered regulation of dopamine release. Parkinsonism Relat. Disord. 21, 1156–1163. 10.1016/j.parkreldis.2015.07.025 26282470

[B204] Volta M. Milnerwood A. J. Farrer M. J. (2015b). Insights from late-onset familial parkinsonism on the pathogenesis of idiopathic Parkinson’s disease. Lancet Neurol. 14, 1054–1064. 10.1016/s1474-4422(15)00186-6 26376970

[B205] West A. B. Cowell R. M. Daher J. P. L. Moehle M. S. Hinkle K. M. Melrose H. L.. (2014). Differential LRRK2 expression in the cortex, striatum, and substantia nigra in transgenic and nontransgenic rodents. J. Comp. Neurol. 522, 2465–2480. 10.1002/cne.23583 24633735 PMC4076169

[B206] West A. B. Moore D. J. Biskup S. Bugayenko A. Smith W. W. Ross C. A.. (2005). Parkinson’s disease-associated mutations in leucine-rich repeat kinase 2 augment kinase activity. Proc. Natl. Acad. Sci. U S A 102, 16842–16847. 10.1073/pnas.0507360102 16269541 PMC1283829

[B207] Winner B. Melrose H. L. Zhao C. Hinkle K. Yue M. Kent C.. (2011). Adult neurogenesis and neurite outgrowth are impaired in LRRK2 G2019S mice B. Neurobiol. Dis. 41, 706–716. 10.1016/j.nbd.2010.12.008 21168496 PMC3059106

[B208] Xiong Y. Neifert S. Karuppagounder S. S. Liu Q. Stankowski J. N. Lee B. D.. (2018). Robust kinase- and age-dependent dopaminergic and norepinephrine neurodegeneration in LRRK2 G2019S transgenic mice. Proc. Natl. Acad. Sci. U S A 115, 1635–1640. 10.1073/pnas.1712648115 29386392 PMC5816154

[B209] Yamada K. Iwatsubo T. (2018). Extracellular α-synuclein levels are regulated by neuronal activity. Mol. Neurodegener. 13:9. 10.1186/s13024-018-0241-0 29467003 PMC5822605

[B210] Ysselstein D. Nguyen M. Young T. J. Severino A. Schwake M. Merchant K.. (2019). LRRK2 kinase activity regulates lysosomal glucocerebrosidase in neurons derived from Parkinson’s disease patients. Nat. Commun. 10:5570. 10.1038/s41467-019-13413-w 31804465 PMC6895201

[B211] Yue M. Hinkle K. Davies P. Trushina E. Fiesel F. Christenson T.. (2015). Progressive dopaminergic alterations and mitochondrial abnormalities in LRRK2 G2019S knock in mice. Neurobiol. Dis. 78, 172–195. 10.1016/j.nbd.2015.02.031 25836420 PMC4526103

[B212] Yun H. J. Kim H. Ga I. Oh H. Ho D. H. Kim J.. (2015). An early endosome regulator, Rab5b, is an LRRK2 kinase substrate. J. Biochem. 157, 485–495. 10.1093/jb/mvv005 25605758

[B213] Yun H. J. Park J. Ho D. H. Kim H. Kim C.-H. Oh H.. (2013). LRRK2 phosphorylates Snapin and inhibits interaction of Snapin with SNAP-25. Exp. Mol. Med. 45:e36. 10.1038/emm.2013.68 23949442 PMC3789260

[B214] Zhao H. T. John N. Delic V. Ikeda-Lee K. Kim A. Weihofen A.. (2017). LRRK2 antisense oligonucleotides ameliorate α-synuclein inclusion formation in a Parkinson’s disease mouse model. Mol. Ther. Nucleic Acids 8, 508–519. 10.1016/j.omtn.2017.08.002 28918051 PMC5573879

[B215] Zhao Y. Dzamko N. (2019). Recent developments in LRRK2-targeted therapy for Parkinson’s disease. Drugs 79, 1037–1051. 10.1007/s40265-019-01139-4 31161537

[B216] Zhou H. Huang C. Tong J. Hong W. C. Liu Y. J. Xia X. G. (2011). Temporal expression of mutant LRRK2 in adult rats impairs dopamine reuptake. Int. J. Biol. Sci. 7, 753–761. 10.7150/ijbs.7.753 21698001 PMC3119847

[B217] Zimprich A. Benet-Pagès A. Struhal W. Graf E. Eck S. H. Offman M. N.. (2011). A mutation in VPS35, encoding a subunit of the retromer complex, causes late-onset parkinson disease. Am. J. Hum. Genet. 89, 168–175. 10.1016/j.ajhg.2011.06.008 21763483 PMC3135812

[B218] Zimprich A. Biskup S. Leitner P. Lichtner P. Farrer M. Lincoln S.. (2004). Mutations in LRRK2 cause autosomal-dominant parkinsonism with pleomorphic pathology. Neuron 44, 601–607. 10.1016/j.neuron.2004.11.005 15541309

